# Anti-Tumor Activity of a *miR-199*-dependent Oncolytic Adenovirus

**DOI:** 10.1371/journal.pone.0073964

**Published:** 2013-09-12

**Authors:** Elisa Callegari, Bahaeldin K. Elamin, Lucilla D’Abundo, Simonetta Falzoni, Giovanna Donvito, Farzaneh Moshiri, Maddalena Milazzo, Giuseppe Altavilla, Luciano Giacomelli, Francesca Fornari, Akseli Hemminki, Francesco Di Virgilio, Laura Gramantieri, Massimo Negrini, Silvia Sabbioni

**Affiliations:** 1 Dipartimento di Morfologia, Chirurgia e Medicina Sperimentale, Università di Ferrara, Ferrara, Italy; 2 Department of Microbiology, Faculty of Medical Laboratory Sciences, University of Khartoum, Khartoum, Sudan; 3 Centro di Ricerca Biomedica Applicata e Dipartimento di Medicina Interna, Policlinico S. Orsola-Malpighi e Università di Bologna, Bologna, Italy; 4 Dipartimento di Scienze Medico Diagnostiche e Terapie Speciali, Università di Padova, Padova, Italy; 5 Cancer Gene Therapy Group, Molecular Cancer Biology Program & Transplantation Laboratory & Haartman Institute, University of Helsinki, Helsinki, Finland; 6 Dipartimento di Scienze della Vita e Biotecnologie, Università di Ferrara, Ferrara, Italy; 7 Department of Molecular Medicine, School of Advanced Technologies in Medicine, Tehran University of Medical Sciences, Tehran, Iran; University of Chicago, United States of America

## Abstract

The down-regulation of miR-199 occurs in nearly all primary hepatocellular carcinomas (HCCs) and HCC cell lines in comparison with normal liver. We exploited this miR-199 differential expression to develop a conditionally replication-competent oncolytic adenovirus, Ad-199T, and achieve tumor-specific viral expression and replication. To this aim, we introduced four copies of miR-199 target sites within the 3’ UTR of E1A gene, essential for viral replication. As consequence, E1A expression from Ad-199T virus was tightly regulated both at RNA and protein levels in HCC derived cell lines, and replication controlled by the level of miR-199 expression. Various approaches were used to asses *in vivo* properties of Ad-199T. Ad-199T replication was inhibited in normal, miR-199 positive, liver parenchyma, thus resulting in reduced hepatotoxicity. Conversely, the intrahepatic delivery of Ad-199T in newborn mice led to virus replication and fast removal of implanted HepG2 liver cancer cells. The ability of Ad-199T to control tumor growth was also shown in a subcutaneous xenograft model in nude mice and in HCCs arising in immune-competent mice. In summary, we developed a novel oncolytic adenovirus, Ad-199T, which could demonstrate a therapeutic potential against liver cancer without causing significant hepatotoxicity.

## Introduction

Oncolytic viruses have been proposed for cancer therapy, since they can be engineered to potentially deliver their cytocidal effect to tumor cells [1,2]. Among them, adenoviruses were widely used as oncolytic viral agents in cancer therapy, as they possess an inherent potential to kill the cells that sustain their replication [3]. However, to restrict cytocidal effect to tumor cells, their replication had to be tightly controlled in normal cells. Hence, conditionally replicative adenoviruses (CRAds) have been developed to restrict viral replication to target cancerous tissues and inhibit replication in normal healthy cells. This has been attempted by exploiting loss-of-function mutations in E1B viral sequences [4], or linking genes E1A/E1B to cancer-specific promoters, such as the telomerase or prostate-specific rat probasin promoters or the human prostate-specific enhancer/promoter [5,6,7]. Additional approaches have also been explored for specific targeting viruses to cancer cells, for example through the introduction of T-cell receptors specific for tumor-specific antigens [8].

More recently, a strategy based on endogenous microRNAs (miRNAs) has been explored to control viral replication [9]. miRNAs are small, non-coding RNA molecules of 20–24 bp that can regulate gene expression at the post-transcriptional level by binding to target transcripts in a sequence-specific manner [10]. These small molecules play a critical role in many cellular processes and several examples of aberrantly regulated miRNAs in human cancer have been reported [11]. Among all, miRNAs expression profiling in hepatocellular carcinomas (HCCs) revealed the existence of differential patterns between tumor tissues and normal liver. In particular, miR-199 was reported to be consistently down-regulated in HCC [12]. The involvement of miR-199 in the pathogenesis of HCC was linked to the abnormal regulation of multiple target genes, such as mTOR, c-Met, HIF-1α and CD44 [13,14,15,16]. Here, we took advantage of this information to produce a new type of CRAd able to replicate only in cells lacking miR-199, with the aim of making viral replication and cytolytic effect specifically selective for HCC cells.

HCC, the fifth most frequent neoplasm and the third leading cause of cancer-related deaths worldwide [17], carries a generally poor prognosis. Complete tumor removal represents the only long-term cure. However, partial hepatectomy can be undertaken in less than 15-30% of patients due to the extent of underlying cirrhosis and, among patients who undergo tumor resection, up to 75% of patients will develop intra-hepatic recurrences within 5 years [18]. When possible, complete hepatectomy and orthotopic liver transplantation (OLT) represents the therapy of choice for patients with significant cirrhosis and limited tumor burden [19,20,21]. In patients who are not candidates for liver transplantation or resection, the most common therapy is transcatheter arterial chemo-embolization (TACE) [22], whose impact on clinical outcome remains unclear. The use of systemic chemotherapy has been attempted but HCC is minimally responsive. More recently, the multi-kinase inhibitor sorafenib, able to target multiple pathways and blocking RAF/MEK/ERK signaling at the level of raf-kinase as well as by inhibiting vascular endothelial growth factor receptor (VEGFR) and platelet-derived growth factor receptor beta (PDGFR-beta), was shown to improve survival and increase time to disease progression in advanced HCCs [23]. In spite of these efforts, with the exception of early and very early tumor stages, HCC remains an incurable disease and new more effective and less toxic therapeutic strategies are needed.

## Materials and Methods

### Ethics statement

The study was carried out in strict accordance with the Guidelines for the Care and Use of Laboratory Animals of the Italian Ministry of Health. Protocol has been approved by the Comitato Etico di Ateneo *per la Sperimentazione Animale* (C.E.A.S.A.) of the University of Ferrara (protocol n. 9752). Animals were sacrificed under inhalational anesthesia with isoflurane to minimize suffering.

### Vectors construction

Four copies of a 22 bp DNA segment complementary to miR199 were inserted within the 3’-untraslated region (3’-UTR) of the E1A gene, which is essential for adenoviral replication. The oligonucleotides containing the sequences (1) 5’-CTA GAT AAC CAA TGT GCA GAC TAC TGT ccT AAC CAA TGT GCA GAC TAC TGT ccT AAC CAA TGT GCA GAC TAC TGT ccT AAC CAA TGT GCA GAC TAC TGT ccT-3’ and (2) 5’-CTA Gag gAC AGT AGT CTG CAC ATT GGT Tag gAC AGT AGT CTG CAC ATT GGT Tag gAC AGT AGT CTG CAC ATT GGT Tag gAC AGT AGT CTG CAC ATT GGT TAT-3’, were synthesized at IDT (Integrated DNA Technologies Inc., Coralville, Iowa, USA), self-annealed and phosphorylated using a polynucleotide kinase (Roche Applied Science, Indianapolis, USA). To confirm its functional activity, we first cloned the miR-199 artificial target sequence downstream of the *firefly* luciferase reporter gene at the XbaI restriction site within the pGL3 vector (Promega, Madison, WI, USA), to generate pGL3/199T vector. This vector was transfected into the hepatocarcinoma Hep3B cells together with either miR-199 or scrambled oligos. As expected, the luciferase activity was significantly decreased only in samples co-transfected with miR-199 (p value = 0.007), thus proving the functional interaction between miR-199 and the artificial target sequences (Figure **S1**). To produce the adenoviral vector, several steps were employed, as depicted in Figure **S2**. The adenoviral backbone was from pAdCMV-V5-Dest (Gateway technology, Invitrogen, Carlsbad, CA, USA), a replication-defective adenovirus that lacks the E1A/E1B locus. The pShuttle/K vector, containing a subgenomic adenovirus type 5 (Ad5) fragment, was the source for the wild type E1A/E1B gene. The E1A/E1B DNA segment included an ectopic translation initiation site in the wrong reading frame within the 5’ UTR of E1A mRNA, to uniformly reduce E1A protein levels in all cell types, and a MluI restriction site within the 3’ UTR of the E1A gene, was previously reported [9]. The MluI site was used for introducing the *miR-199* target segment, originally cloned into the pGL3/199T (as described above). The non-replicative adenovirus Ad-Null-Control (ADV-001) was provided as a premade Recombinant Adenovirus from Cell Biolabs (Cell Biolabs, Inc, San Diego, CA).

To produce the pIRES-miR199 plasmid, a 650 bp region of human genomic DNA including pre-miR and mature miRNA sequence was amplified from 293 cells with the primers miR199NheI_Fwd (5’-GCT AGC GAC CCC CAA AGA GTC AGA CA-3’) and miR199NheI_Rev (5’-GCT AGC CCA CCC TCT TAG ATG CCT CA-3’). The fragment was cloned into a pIRESneo2 plasmid at the NheI restriction site and controlled by sequencing. The pIRES-Luc plasmid was constructed cloning the *Luciferase* gene (obtained from a pGL3-Control vector, Promega, Madison, WI, USA) in a pIRESneo2 backbone at a NheI restriction site.

### Cell culture

The hepatocellular carcinoma cell lines HepG2 (ATCC HB-8065) and Hep3B (ATCC HB-8064) were obtained from the American Type Culture Collection (ATCC, Manassas, VA). The human embryonic kidney cells, 293FT transformed with the SV40 large T antigen were obtained from Invitrogen (Carlsbad, CA, USA). Cell lines were propagated and maintained in Dulbecco’s Modified Iscove’s Medium (IMDM) supplemented with 10% fetal bovine serum (FBS), 0.1% Gentamycin and 1% L-glutamine (Sigma, St Louis, MO).

### Luciferase Assay

Luciferase expression was analyzed using the Dual Luciferase Reporter Assay (Promega, Madison, WI, USA), following the manufacturer’s protocol. Hep3B cells were plated at a density of 7x10^4^ cells/well in a 24 wells plate. Transfections were performed with 400 ng of pGL3/199T vector (firefly luciferase) and normalized by co-transfecting 40 ng of pRL-TK (renilla luciferase) vector. The pre-miR-199a-3p miRNA precursor (Ambion Applied Biosystems, Grand Island, NY, USA) or the control oligonucleotide (AM17111, Ambion Applied Biosystems, Grand Island, NY, USA) were co-transfected at a concentration of 100 nM.

### HepG2 derived cell lines

To produce stable cell clones expressing miR199, HepG2 cells were transfected with 2 µg of a miR-199 expressing plasmid, pIRES-miR199, using Lipofectamine 2000 (Invitrogen, Carlsbad, CA). To produce stable cell clones expressing the *firefly* Luciferase reporter gene, HepG2 cells were transfected with 2 µg of a Luciferase expressing vector, pIRES-Luc. After 24 hours, cells were diluted into T75 flasks and subjected to selection using 700 µg/ml G418 (Roche Applied Science, Indianapolis, USA) for 2 weeks.

### Real-Time RT-PCR analysis

The expression of mature miRNAs was quantified by the Taqman MicroRNA Assays (Applied Biosystems, Grand Island, NY, USA). Total RNA was isolated from cells using Trizol reagent (Invitrogen). Reverse transcription reaction was done starting from 5ng of total RNA. Real-Time PCR was performed using the standard Taqman MicroRNA Assay protocol on the iCycler iQ Real-Time PCR Detection System (Biorad). The reactions were incubated at 95°C for 10 min followed by 40 cycles of 95°C for 15s and 60°C for 1 min. The ∆∆Ct method for relative quantification of gene expression was used to determine miRNA expression levels. Fold change was generated using the equation 2^-∆∆Ct^. Each sample was analyzed in triplicate. To normalize the relative abundance of miRNA was used the Taqman Assays for U6 RNA (RNU6B, Applied Biosystems, Grand Island, NY, USA).

### Production of Recombinant Adenoviruses

Recombinant adenovirus encoding the E1A/199T/E1B region and the IRES-EGFP cassette expressing the green fluorescent protein was produced using the *in vitro* recombination techniques. Briefly, the E1A/E1B region for the adenovirus genome (GenBank, accession number AC_000008) was amplified by PCR from the plasmid pShuttle/K and subcloned into a pGEM-T vector (Promega, Madison, WI, USA). The fidelity of the clone was verified by sequencing. For the construction of the adenoviral control vector, the EcoRI E1A/E1B fragment was excised from the plasmid pGEM_E1A/E1B and cloned into the EcoRI site of a modified pENTR11 vector (Invitrogen, Carlsbad, CA, USA) containing an IRES/EGFP sequence, to generate the plasmid pENTR_E1A/E1B. For the construction of the adenoviral miR-199 dependent plasmid, an MluI fragment containing the microRNA-199 target sequence was amplified by PCR from the plasmid pGL3-199T and inserted into a MluI site located in the 3’ UTR of E1A sequence in the vector pENTR_E1A/E1B, to generate pENTR_E1A/199T/E1B. To construct recombinant plasmids, the ViraPower Adenoviral Gateway Expression System was used, according to the manufacturer’s instructions (Invitrogen, Carlsbad, CA, USA). The recombinant adenoviral plasmids obtained, pAd-199T and pAd-Control, were linearized with PacI restriction enzyme and then transfected into 293FT cells using Lipofectamine 2000 (Invitrogen, Carlsbad, CA, USA). The viral progeny were packaged into 293FT cells and purified using PEG-it Virus Precipitation Solution (System Bioscences, Mountain View, CA), according to manufacturer’s instructions.

### Titration of Recombinant Adenoviruses

To determine the titer of Ad-199T and Ad-Control viruses, 7x10^4^ HepG2 cells were seeded in a 24 well plate; after 24 hours, cells were infected with 1 microliter of either viruses. One day after infection, cells were harvested and total genomic DNA was purified by QIAmp DNA Mini Kit (Qiagen, Hilden, Germany) according to manufacturer’s instructions. 50ng of genomic DNA was analyzed by quantitative Real-Time PCR, using EVA green (Biotium Inc, Hayward, CA, USA) and primers specific for wild type Ad5 sequences: wtAd5Fwd, 5′- CGC ATA CGA GCA GAC GGT GAA C-3′; wtAd5Rev, 5′- GCA CTA TAA GGA ACA GCT GCG CC -3′. PCR was performed by initial denaturation at 95°C for 15min followed by 40 cycles of 30s at 95°C, 30s at 58°C and 30s at 72°C. A standard curve was generated by using serial dilutions of the plasmid pAd-Control DNA (from 10ng to 100fg) spiked in 50 ng of HepG2 non infected genomic DNA. The number of viral infectious units (I.U.) was determined comparing the threshold cycle (Ct) values of each sample with those of standard samples. Fluorescence was measured by using a Biorad-Chromo4 thermal cycler real-time PCR instrument.

### Assessment of Viral Replication

To assess viral replication, adenoviral DNA in infected cells was quantified by Real-Time PCR, using EVA green (Biotium Inc, Hayward, CA, USA) and primers wtAd5Fwd and wtAd5Rev, specific for wild type Ad5. PCR was performed by initial denaturation at 95°C for 15min followed by 40 cycles of 30s at 95°C, 30s at 58°C and 30s at 72°C. Fluorescence was measured by using a Biorad-Chromo4 thermal cycler real-time PCR instrument. Human or mouse β-actin gene was used to normalize data. Primers were as follows: Hβ-actinDNA2810Fwd, 5’-AGC TGT CAC ATC CAG GGT CC -3’; Hβ-actinDNA2960Rev 5’- TCA TAC TCC TGC TTG CTG ATC C -3’, Mβ-actinDNAFwd, 5’- AAG GGT TAC CCG GGA TAC TG-3’; Mβ-actinDNARev, 5’- TGT TAC CAA CTG GGA CGA CA-3’.

### Analytical PCR on genomic DNA

Genomic DNA from infected cells and from frozen tissues was extracted with the QIAmp DNA Mini Kit (Qiagen, Hilden, Germany) according to manufacturer’s instructions. Analytical PCR, was performed by initial denaturation at 95°C for 15min followed by 30 cycles of 30s at 95°C, 30s at the specific annealing temperature and 30s at 72°C. Viral DNA was analyzed using primers wtAd5Fwd and wtAd5Rev, specific for wild type Ad5. For human TPEF detection, primers were as follows: TPEF-Fwd, 5'-CTC AGC GGA CGA CCC TCT CGC TCC G-3'; TPEF-Rev, 5'-GGC GGC GGC GGT GGC AGT GG-3'. Human or mouse β-actin gene was used to normalize data.

### Adenovirus E1A gene expression

Quantification of Adenovirus E1A mRNA expression was carried out using EVA Green-based Real-Time PCR detection. Total RNA was extracted from a portion of frozen liver tissues after homogenization, with Trizol reagent (Invitrogen) according to manufacturer’s instructions. For quantitative PCR analysis, 200ng of purified RNA were retro-transcribed and 5 µl of cDNA was used for the PCR reaction using EVA Green (Biotium Inc, Hayward, CA, USA). All reactions were carried out in a 20 microliters. Primers were as follows: E1A_1325Fwd, 5’-CCC GAC ATC ACC TGT GTC TA-3’ and E1A_1465Rev, 5’-GAT ACA TTC CAC AGC CTG GC-3’; 18sFwd, 5′-AGC AGC CGC GGT AAT TCC AGC T-3′ and 18sRev, 5′-CGG GAC ACT CAG CTA AGA GCA TC-3′. PCR was performed by initial denaturation at 95°C for 15min followed by 40 cycles of 30s at 95°C, 30s at 58°C and 30s at 72°C. The threshold cycle (Ct) values of each sample were used in the post-PCR data analysis. Each sample was analyzed in triplicate. E1A expression levels were normalized against ribosomal RNA 18S, as housekeeping gene. Fluorescence measurements were completed using a Biorad-Chromo4 thermal cycler real-time PCR instrument.

### Western Blot analysis

To evaluate the expression of adenoviral E1A protein, cells were seeded in 24-well plates at a density of 7×10^4^ cells/well, cultured for 24 h, and infected with 1×10^6^ I.U of Ad-Control or Ad-199T. Two days later, cells were harvested and lysed by using RIPA lysis buffer (150 mM NaCl, 0.1% SDS, 0.5% sodium deoxycholate, 1% NP-40) (Sigma, St Louis, MO) with complete protease inhibitor cocktails (Sigma, St Louis, MO). Homogenates were then centrifuged at 13000 rpm for fifteen minutes at 4°C and supernatants were collected and analyzed by Western blot to assess E1A protein expression with a polyclonal anti-E1A antibody (sc-430, Santa Cruz Biotechnology, Santa Cruz, CA, USA). Digital images of autoradiographies were acquired with Fluor-S MultiImager (BioRad) and band signals were acquired in the linear range of the scanner using the densitometric module of the software Quantity One (BioRad). After autoradiography acquisition, the membranes were reprobed for 1 h at room temperature with anti-β-tubulin polyclonal antibody H-235 (sc-9104, Santa Cruz Biotechnology, Santa Cruz, CA) diluted 1:1000 as housekeeping gene.

### Immunohistochemical analysis

A rabbit polyclonal lgG anti-phospho-H2A.X (Upstate, Cell Signaling Solutions, NY, USA) recognizing the phosphorylated Ser 139 of histone H2A.X was used as primary antibody for immunohistochemistry. Immunoreactivity was revealed with the HRP (Horse-Radish-Peroxidase) EnVision system (DAKO, Denmark), and DAB (diaminobenzidine) as chromogen (Sigma, St Louis, USA). Primary antibody was incubated over night at 1:200 dilution. The secondary antibody was incubated 1 hour at room temperature. Mounted slides were examined by light microscopy and immunoreactivity assessed by using a 3-grade system: 0, no staining; +, minimal staining and ++, uniform or intense staining.

### *In vivo* mouse experiments

For the treatment of liver cancer, the miR-221 transgenic strain (TG221), which is predisposed to the development of liver cancer, was employed [24]. This mouse strain has an increased susceptibility to the carcinogen diethylnitrosammine (DEN) and tumors exhibit a miRNA profile similar to human HCC, including the down-regulation of miR-199. The mice were maintained in a vented cabinet at 25°C with 12-hour light-dark cycle and provided food and water ad libitum. To facilitate tumor development, DEN was injected intra-peritoneally (7.5 mg/kg body weight) at day 10 after birth. Virus injections into the tail vein were performed by using 1x10^8^ I.U. of Ad-199T or control virus. All mice were sacrificed at the end of month 5, subjected to autopsy. Livers were partly fixed in 10% formalin for histopathological investiagations and partly frozen in liquid nitrogen for molecular studies. Liver DNA was isolated using the QIAmp DNA Mini Kit (Qiagen, Hilden, Germany) and RNA by using with Trizol reagent (Invitrogen), according to the manufacturers’ procedures. For “nude” mice experiments, mice were maintained in a vented cabinet at 30°C with 12-hour light-dark cycle and provided food and water ad libitum. Hep3B tumor xenografts were established by subcutaneous inoculation of 5x10^6^ cells into the right flank of 4 week-old CD-1 nude mice (Charles River Laboratories International, Wilmington, MA), maintained at pathogen-free conditions. When tumors reached 5 to 15 mm^3^, the animals were randomized in 2 groups (n=6 mice per group) and treated with either phosphate buffered saline (PBS) solution or 5x10^8^ I.U. Ad-199T virus (diluted into PBS) by intra-tumoral injection. The injections were performed once every other day for a total of six injections. Tumors were measured every 2 days and volume was calculated by the formula (length x width^2^)/2. Animals were sacrificed when the tumor volume reached 1,000 mm^3^, which was also the end-point of the overall survival analysis.

### *In Vivo* Imaging System (IVIS)

In vivo bioluminescent imaging was performed with a ultra-low noise, high sensitivity cooled CCD camera, mounted on a light tight imaging chamber (IVIS 100 SystemTM, Xenogen, Roissy, France). Tracking, monitoring and quantification of signals were controlled by the acquisition and analysis software Living ImageH (Xenogen Corp, Alameda, CA). D-luciferin was injected intra-peritoneum (i.p.) at a dose of 150 mg/kg body weight (30 mg/ml luciferin) to anesthetized (1–3% isoflurane) animals 15 minutes before image acquisition. Anesthetized mice were then placed in the IVISTM Imaging System and imaged. Three-four mice were imaged at each time. Regions of interest from displayed images were identified around the tumor sites and were quantified as total photon counts or photons/s using the Living ImageH software (Xenogen Corp, Alameda, CA).

### Statistical analysis

Statistical significance of groups similarity was resolved using a 2-tails Student’s t test. A p-value threshold < 0.05 was considered significant. When appropriate, group value was expressed as mean ± standard deviation (SD). Survival analysis was performed by using a Kaplan-Meier plot, and significance assessed by the log rank test.

## Results

### Construction of a replication selective adenovirus

**Figure 1:**
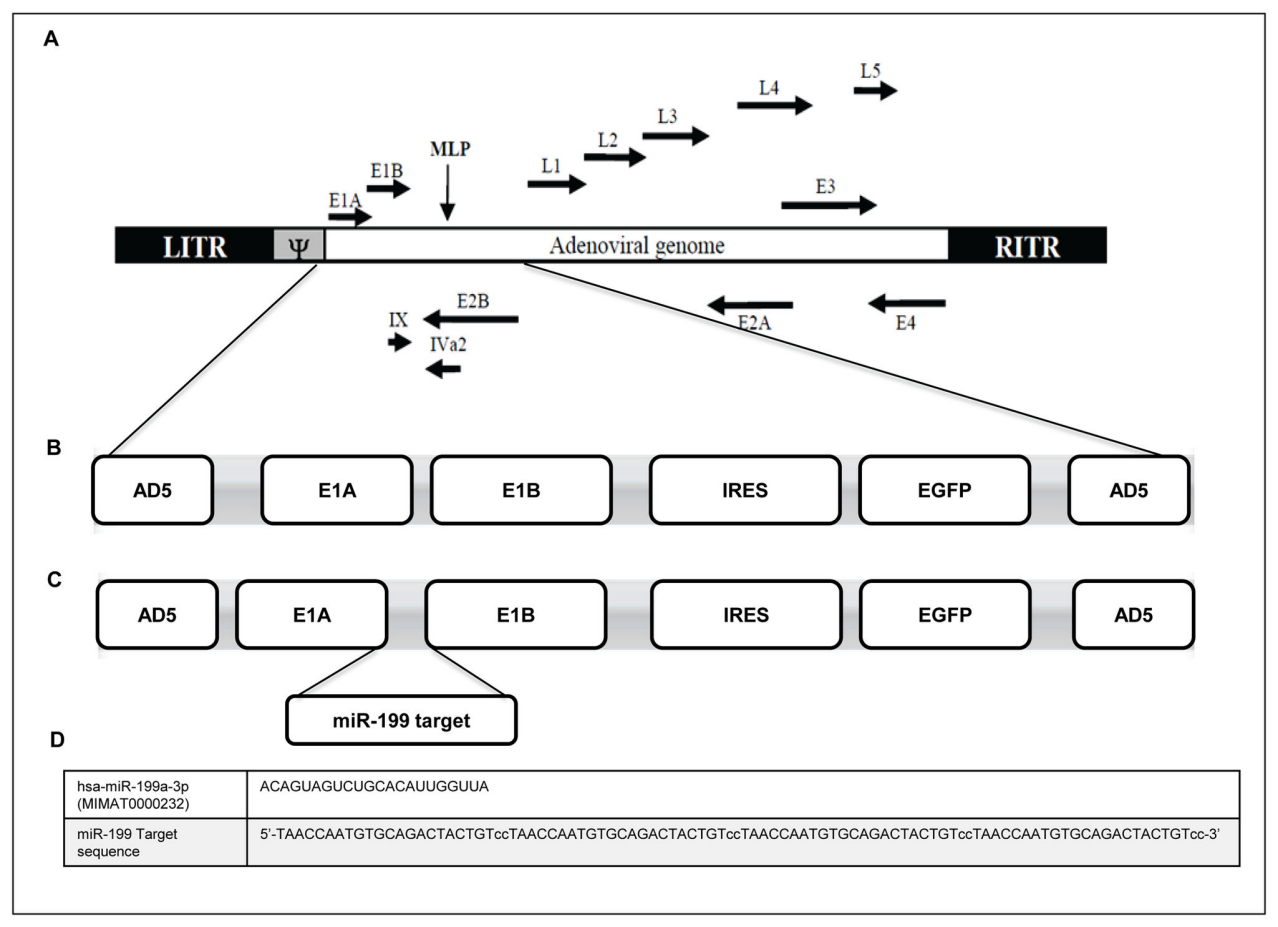
Description of the conditionally replication-competent oncolytic Adenovirus. (**A**) Schematic illustration of Adenovirus type 5 genome: the viral early and late genes and the Inverted Terminal Repeat (ITR) are indicated. (**B**) TheAd-Control viral genome encloses the E1A/E1B genes and the IRES-EGFP expression cassette. (**C**) The Ad-199T viral genome contains a specific sequence complementary to microRNA 199a-3p (miR-199 target) in the 3’ untranslated region of the E1A gene. (**D**) The miR-199 target sequence is made of 4 copies of a 22bp DNA segment complementary to the mature miRNA.

The rationale of the work was based on the differential expression of miR-199 between normal versus cancer liver cells and in particular on the basis that miR-199 is down-regulated in human hepatocellular carcinoma [12]. To develop a conditionally replication-competent oncolytic adenovirus (CRAd) under miR-199 control, named Ad-199T, four copies of a 22 bp DNA segment complementary to human and mouse miR-199, were inserted within the 3’-untraslated region (3’-UTR) of the E1A gene, essential for adenoviral replication. For the construction of the adenoviral vectors, the adenoviral backbone was from pAdCMV-V5-Dest (Invitrogen), which lacks the E1A/E1B locus. The E1A/E1B sequences were obtained from the pShuttle/K vector [9] by PCR amplification using primers containing EcoRI sites, and introduced within the EcoRI site of the intermediate vector pENTR-IRES-EGFP. A replicative control adenovirus, Ad-Control, without miR-199 target sequences, was also developed (Figure **1**). Details of vectors construction are described in Materials and Methods and in Figure **S2**. The titer of the produced adenoviruses were as follows: 6.24x10^10^ I.U/ml pAd-199T and 1.95x10^10^ I.U/ml pAd-Control.

### Ad-199T replication is microRNA-dependent

**Figure 2:**
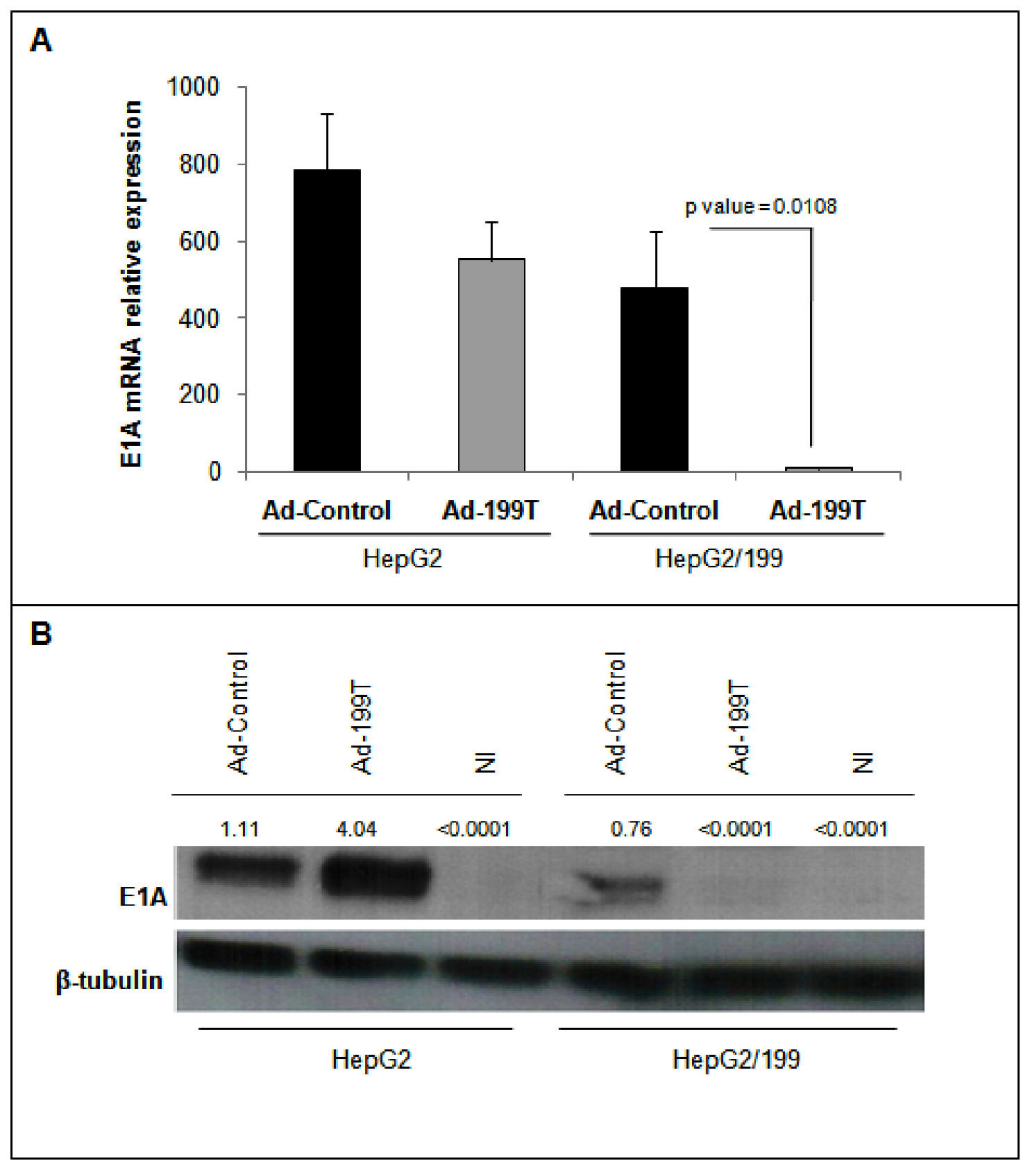
E1A expression is miR-199 dependent. To assess the expression of E1A in presence or absence of miR-199, 7×10^4^ cells of HepG2 and HepG2/199 cell lines were seeded and infected with 1x10^6^ I.U. of Ad-199T or with 1x10^6^ I.U. of Ad-Control and harvested after 48 hours. E1A levels were evaluated by quantitative PCR analysis (**A**) and by Western Blot (**B**). In HepG2, E1A mRNA was expressed following infection by both viruses; conversely, in HepG2/199 the E1A mRNA levels were significantly lower in AD-199T infected cells compared to Ad-Control infected cells. P-values of the comparisons are shown. The same results were obtained at the protein level. This evidence confirms that miR-199, constitutively expressed in HepG2/199 cells, can control E1A expression by targeting the homologous sequences places downstream of the adenoviral E1A gene.

**Figure 3:**
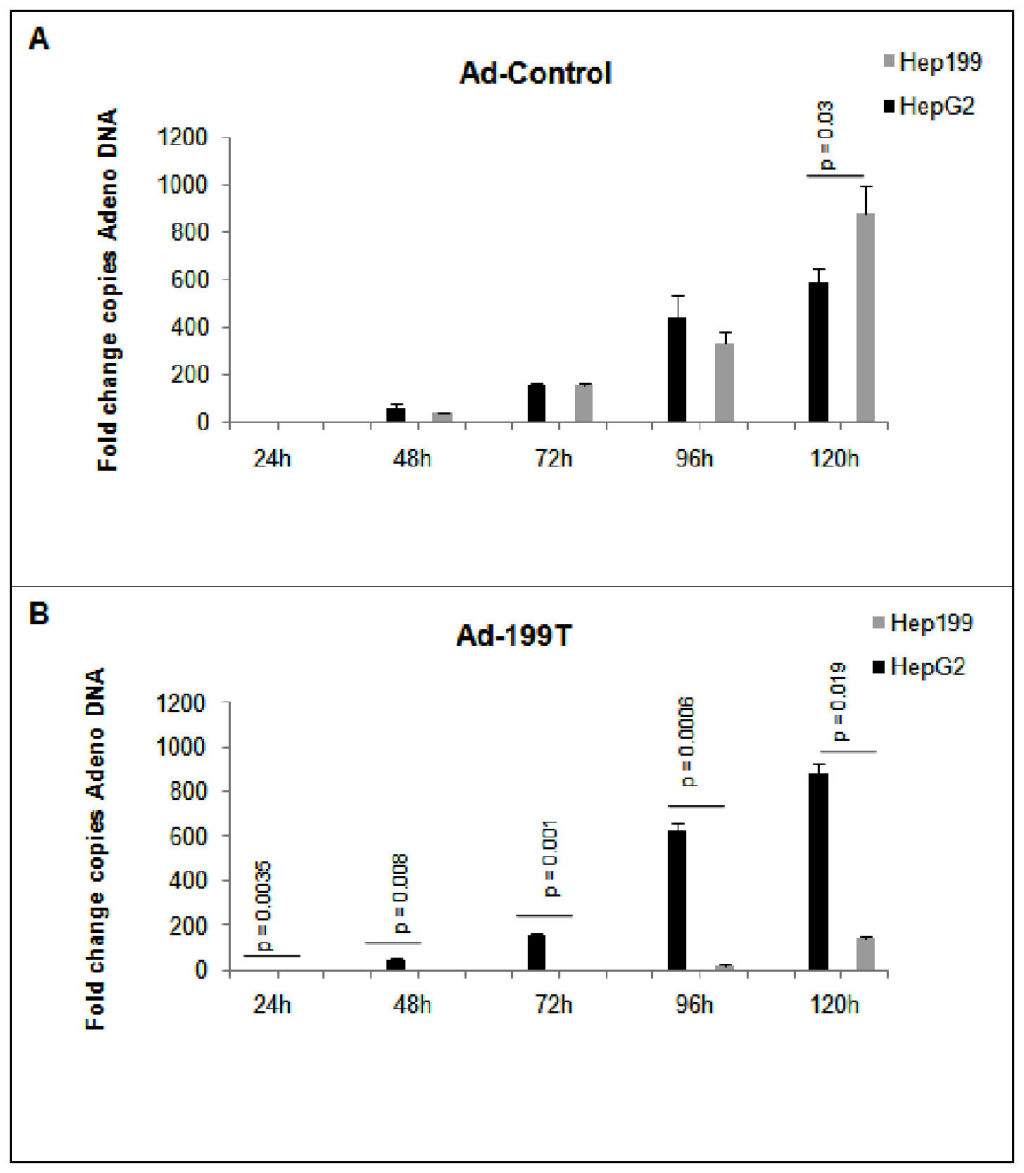
miR-199 controls Ad-199T replication *in vitro*. (**A**-**B**) To asses viral replication in absence and in presence of miR-199, HepG2 and HepG2/199 cell lines were infected with Ad-199T or Ad-Control adenoviruses. A progressive accumulation of viral DNA, indicated as fold change copies of Adeno DNA referred the lower level of Adenovirus DNA copies, indicates that active viral replication is efficiently occurring in both cell lines infected with Ad-Control virus. A progressive accumulation of viral DNA in the HepG2 cells infected with Ad-199T indicates that active viral replication is efficiently occurring in this cell line; on the contrary, in HepG2/199 cells infected with Ad-199T the viral DNA did not increase over time, confirming that the virus replication was inhibited by the presence of miR-199. P-values of the comparisons are shown.

**Figure 4:**
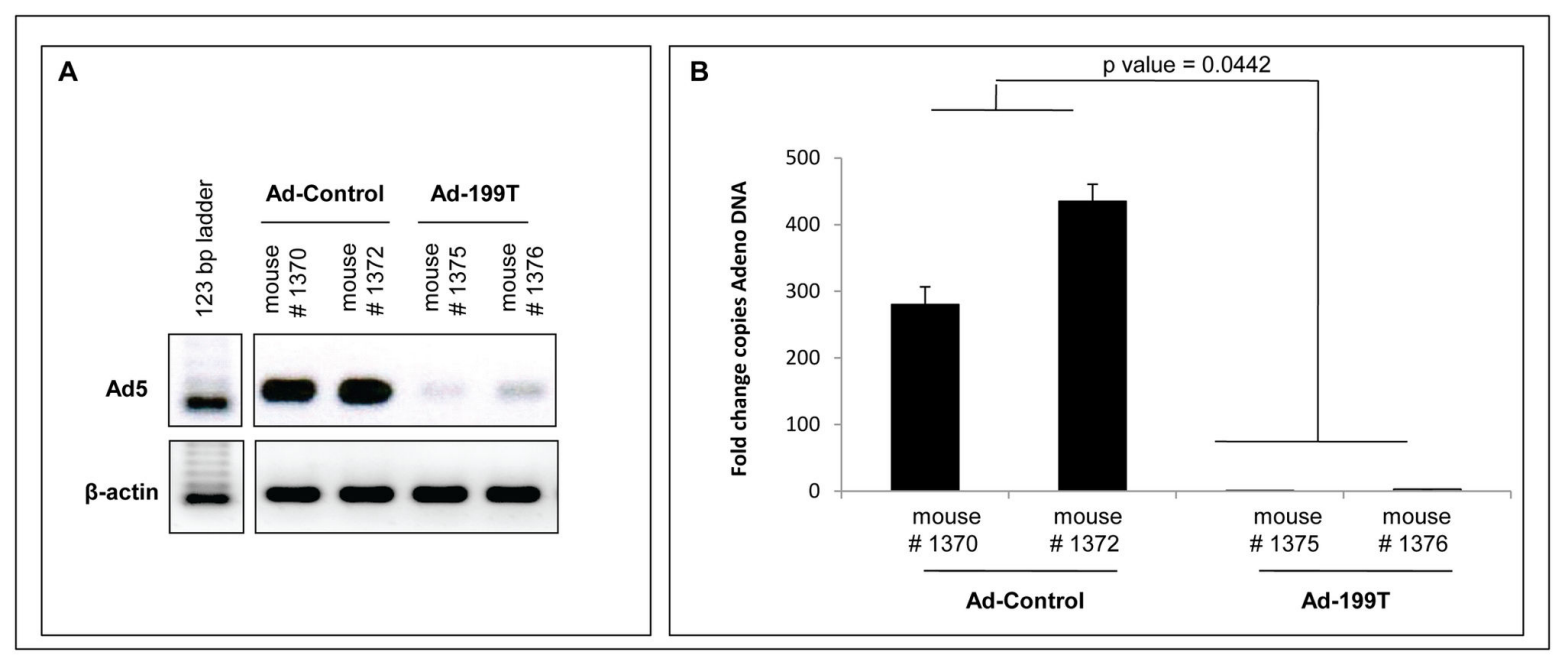
miR-199 controls Ad-199T replication *in vivo*. To assess replication properties of AD-199T *in* *vivo*, 1x10^8^ I.U. of Ad-199T or 1x10^8^ I.U. of Ad-Control were intra-hepatically injected into 3 days old B6D2 mice. At 72 hours after infection, livers were collected and viral DNA quantified using analytical PCR (**A**) and quantitative PCR as fold change copies of Adeno DNA referred to the lower level of Adenovirus DNA copies (**B**) The replication of Ad-199T (mice 1375, 1376) was significantly suppressed in normal liver comparing with Ad-Control (mice 1370, 1372). This evidence confirms that miR-199 could control Ad-199T replication in normal liver cells *in* *vivo*.

To verify if miR-199 could regulate viral replication *in vitro*, Ad-199T and Ad-Control were used to infect two different cell lines: (1) HepG2, derived from human liver carcinoma and not expressing miR-199; (2) HepG2/199, which derives from HepG2 cells engineered to constitutively express miR-199a (Figure **S3**). To this purpose, 7×10^4^ cells of each cell line were seeded and infected with 1x10^6^ I.U. of Ad-199T or with 1x10^6^ I.U. of Ad-Control. The cells were harvested after 24, 48, 72, 96 and 120 hours to assess E1A viral gene expression together with viral replication. The inhibition of E1A mRNA and protein was demonstrated in miR-199 expressing HepG2 cells, while E1A normal expression could be detected in HepG2 wild type cells (Figure **2A-B**). Correspondingly, the active viral replication occurred for both viruses in HepG2 cells, while in HepG2/199 cells only Ad-Control could replicate and Ad-199T virus was inhibited (Figure **3**). These results established that viral replication of Ad-199T was indeed miR-199-dependent *in vitro*. To assess replication properties of Ad-199T *in vivo*, we tested its ability to replicate in the liver of B6D2 wild type mice, where miR-199 is constitutively expressed. To this purpose, 1x10^8^ I.U. of Ad-199T virus or 1x10^8^ I.U. of Ad-Control virus were intra-hepatically injected into 3 days old mice. At 72 hours after infection, livers were collected and genomic DNA was extracted as described in methods section. Viral DNA was quantified by qPCR using primers specific for Adeno-5 wild type sequence. The results demonstrated that the viral DNA was significantly reduced in livers of mice infected with Ad-199T virus in comparison with livers of mice treated with Ad-Control (p-value = 0.0442) (Figure **4**). Effect of viral treatment on normal liver was also evaluated by histological analyses. No significant histopathological changes were detectable in Ad-199T treated livers, with a well preserved liver architecture and nearly absent necrotic damage. On the other side, Ad-Control induced significant hepatotoxicity: Ad-Control treated livers were characterized by a poorly preserved liver architecture, with portal tracts barely distinguishable due to hepatocyte swelling. Hepatocytes enlargement was associated with nuclear dissolution, indicative of necrotic damage. Large areas with hepatocytes with macro- and micro-vesicles were visible. In addition, immuno-staining for the phosphorylated form of the histone H2AX, an early marker of double strand breaks, displayed a nearly complete staining of the nuclei following infection of Ad-Control, but absent in Ad-199T-infected livers (Figure **S4**). These results demonstrated that Ad-Control replicates efficiently in normal liver cells, inducing hepatotoxicity, while miR-199 could control Ad-199T lytic cycle in normal hepatocytes in vivo*.*

### Ad-199T can eliminate tumor cells with same efficiency of Ad-Control virus

**Figure 5:**
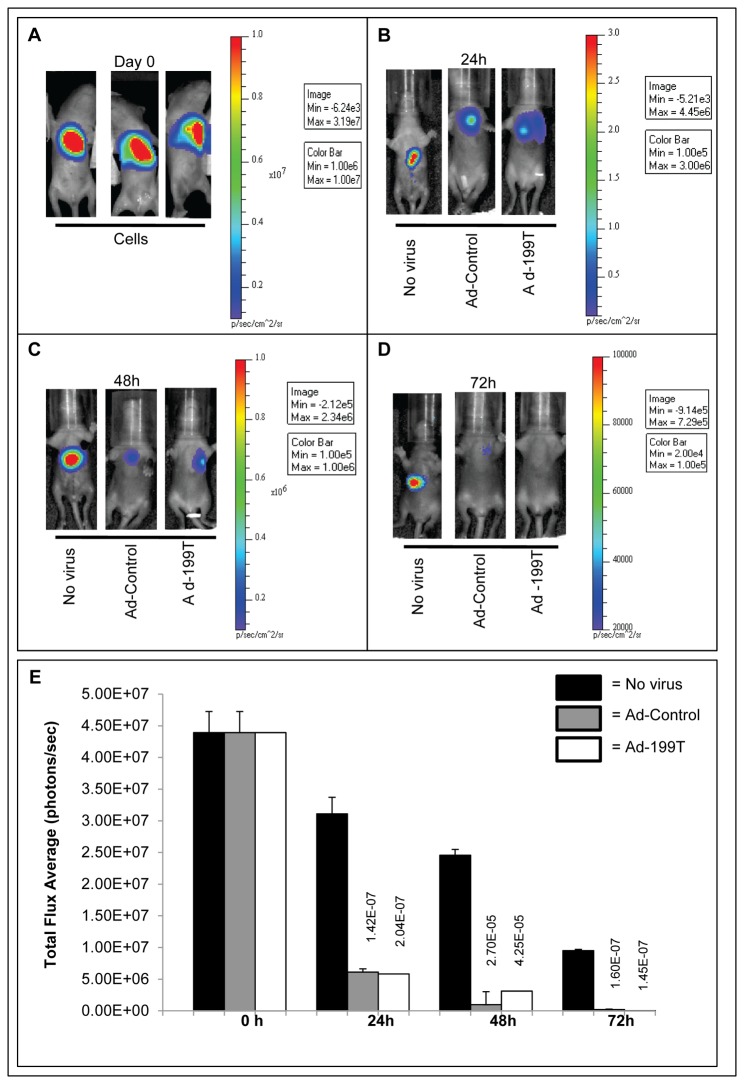
Ad-199T replicates in tumor cells *in vivo*. (**A**) 2x10^6^ HepLuc cells were intra-hepatically implanted in B6D2 wild type mice at 3 days of birth and examined at the In Vivo Imaging System (IVIS) two hours after cells implantation. After 24 hours, three experimental groups, consisting of six mice each, were defined: one was injected intra-hepatically with 1x10^8^ I.U. of the Ad-199T virus; one was infected with 1x10^8^ I.U. of the Ad-Control virus; one was not infected (no virus). Mice were monitored at the IVIS at 24h (**B**), 48h (**C**) and 72h (**D**) after virus inoculation. Reduction in pseudo-color images, representing bioluminescence intensity, indicates that the implanted tumor cells were eliminated, likely due to the active viral replication by either Ad-Control or Ad-199T viruses. (**E**) A quantitative photon analysis of the region-of-interest showed a highly significant decrease (p value <0.05) of luminescence in mice infected with Ad-Control and Ad-199T viruses versus uninfected animals.

After proving that Ad-199T virus can poorly replicate in normal liver cells, we investigated whether this same virus could instead replicate and have a cytocydal effect in tumor cells *in vivo*. To this aim, 2x10^6^ HepLuc cells, HepG2 cells engineered to express the *Firefly* luciferase reporter gene (Figure **S5**), were implanted into the liver of 3 days old B6D2 wild type mice. To verify the presence of the HepLuc cells into the target liver tissue, the mice were examined at the In Vivo Imaging System (IVIS) Spectrum and light emission measured two hours after cell implantation. The detection of a strong light emission signal established the presence of implanted cells in the liver of all animals (Figure **5A**). After 24 hours, three experimental groups, consisting of six mice each, were defined: one was infected intra-hepatically with 1x10^8^ I.U. of Ad-199T virus; the second with 1x10^8^ I.U. of Ad-Control virus; the third group was not infected and used as control to monitor HepLuc cells luminescence during the experimental time-frame. Mice were monitored at the IVIS at 24, 48 and 72 hours after virus infection. Non infected animals exhibited a strong signal at 24 hours, which progressively and gradually decreased at 48 and 72 hours, indicating the presence of the cells during all the observation time points. Conversely, in the virally infected animals the signal decreased more rapidly, to almost completely disappear at 72 hours. These results suggested that the implanted tumor cells were likely eliminated due to active viral replication by either Ad-Control or Ad-199T (Figure **5B-D**). A quantitative photon counting analysis of the region-of-interest showed a highly significant decrease (p-value <0.05) of luminescence in mice infected with the Ad-Control or the Ad-199T viruses vs. uninfected control animals (Figure **5E**). At 72 hours, HepLuc cells derived liver tumor mass was evident in correspondence of the signal detected at the IVIS luminometer in non-infected animals. Conversely, in mice injected with both viruses, there was evidence of significant reduction or loss of liver tumor masses, consistent with the absence of luminous signal (Figure **S6**). These data indicated that both Ad-199T and Ad-Control viruses could replicate *in vivo* and eliminate the implanted tumor cells. To exclude that antitumor effect was prevalently induced by an immune response against adenovirus antigens, we included a group of animals injected with a replication-defective adenovirus (Figure **S8**). The experiment showed that, while an immune response to virus antigens was also involved in tumor cells removal, the lytic cycle of the virus was still producing the fastest removal of tumor cells. The difference of light emission between cells infected with replication-competent versus replication-defective viruses remained statistically significant at all time-points.

**Figure 6:**
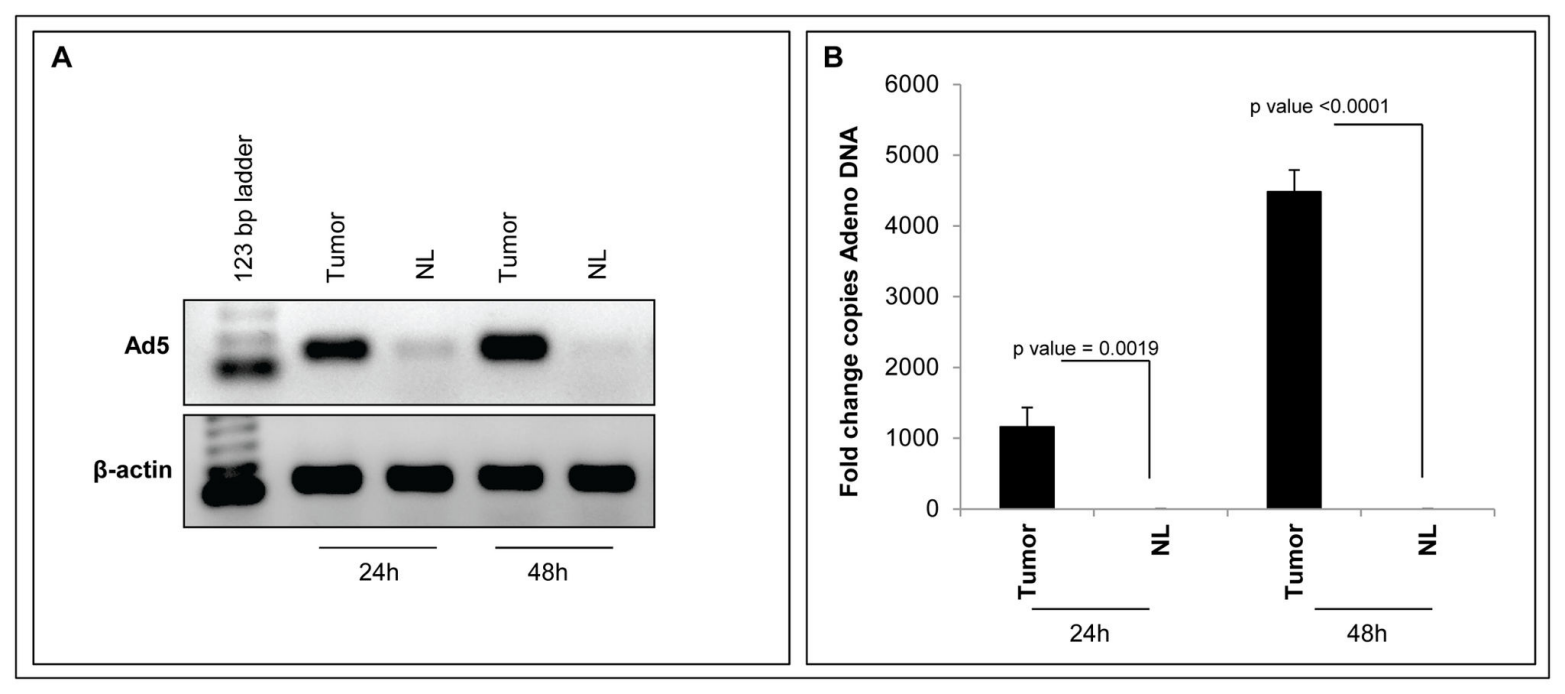
Differential replication of Ad-199T in normal liver versus tumor cells. 2x10^6^ HepLuc cells were intra-hepatically implanted in six B6D2 wild type mice 3 days after birth. After 24 hours, the animals were injected intra-hepatically with 1x10^8^ I.U. of the Ad-199T virus. Mice were sacrificed at 24h and 48h after the treatment and livers as well as tumor cells masses were collected. Genomic DNA extracted both from normal liver and tumor masses was analyzed by analytical PCR (**A**) and quantitative PCR as fold change copies of Adeno DNA referred to the lower level of Adenovirus DNA copies (**B**). The results show a significant suppression of Ad-199T replication in normal liver (NL) compared to tumors (Tumor). P-values of the comparisons are shown.

To assess viral replication and confirm that Ad-199T replication was restricted to cancer cells, HepLuc cells were implanted into the liver of a new group of B6D2 wild type mice and treated with 1x10^8^ I.U. of Ad-199T virus after 24 hours. The animals were sacrificed at 24 and 48 hours after the treatment and livers were collected for DNA analyses: the presence of human HepLuc cells derived DNA was confirmed in the tumors by analytical PCR (Figure **S7**) and then the presence of viral DNA was assessed both in the tumors and in the surrounding normal parenchima. The presence of viral DNA was detected only in tumor tissues, indicating that an active viral replication was occurring in neoplastic tissue but not in normal liver (Figure **6**). The result confirms that the replication of Ad-199T virus was significantly suppressed in normal liver compared to tumor cells.

### Ad-199T has antitumor efficacy in a HCC implanted tumor model

**Figure 7:**
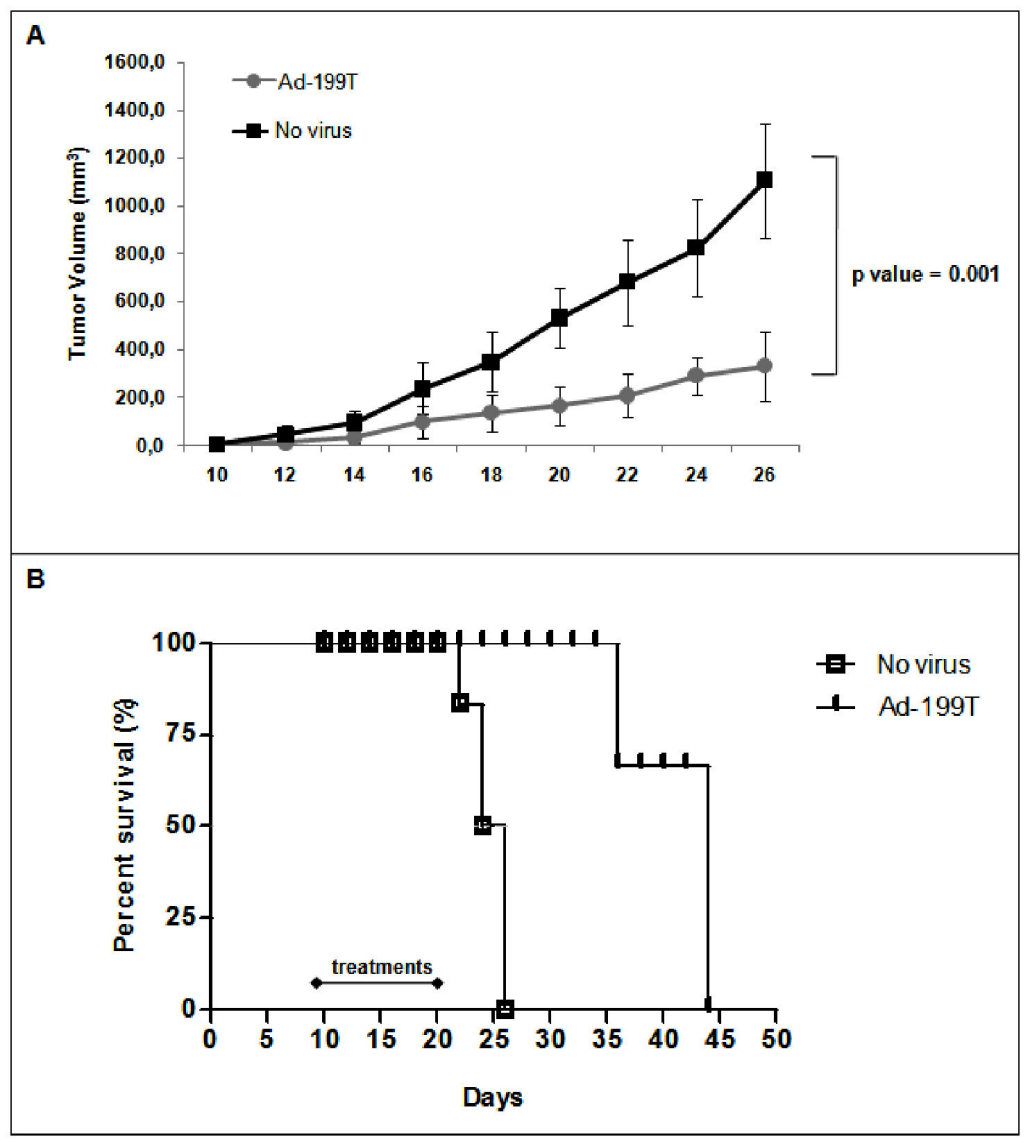
Ad-199T antitumor activity on HCC xenograft. CD1 nude mice (n=6) bearing Hep3B xenografts were treated intra-tumorally either with PBS or with Ad-199T (5x10^8^ I.U. each treatment, for a total of six). (**A**) A tumor growth curve was built by measuring the size of tumors every two days. The results shown a significant difference between the PBS-treated group and the Ad-199T-treated one (p=0.001), confirming the antitumor activity of Ad-199T virus. (**B**) Kaplan-Meier survival plot showed a median survival of 24 days for untreated animals and 45 days for Ad-199T treated animals, thus indicating a longer survival time in animals treated with the oncolytic adenovirus. This difference was highly significant according to the log-rank test (p <0.0001).

The antitumor potential of Ad-199T virus was evaluated in a subcutaneous xenograft of Hep3B tumor cells. Ad-199T replication in Hep3B cells was preliminarily verified *in vitro* (Figure **S9**). Then, nude mice bearing Hep3B tumors were intra-tumorally treated three times a week for two weeks either with phosphate buffer or with Ad-199T virus (5x10^8^ I.U. each injection). The group of Ad-199T treated mice exhibited a kinetic of tumor growth significantly reduced (Figure **7A**), which resulted in an increased survival (Figure **7B**).

### Ad-199T can control liver tumorigenicity

**Figure 8:**
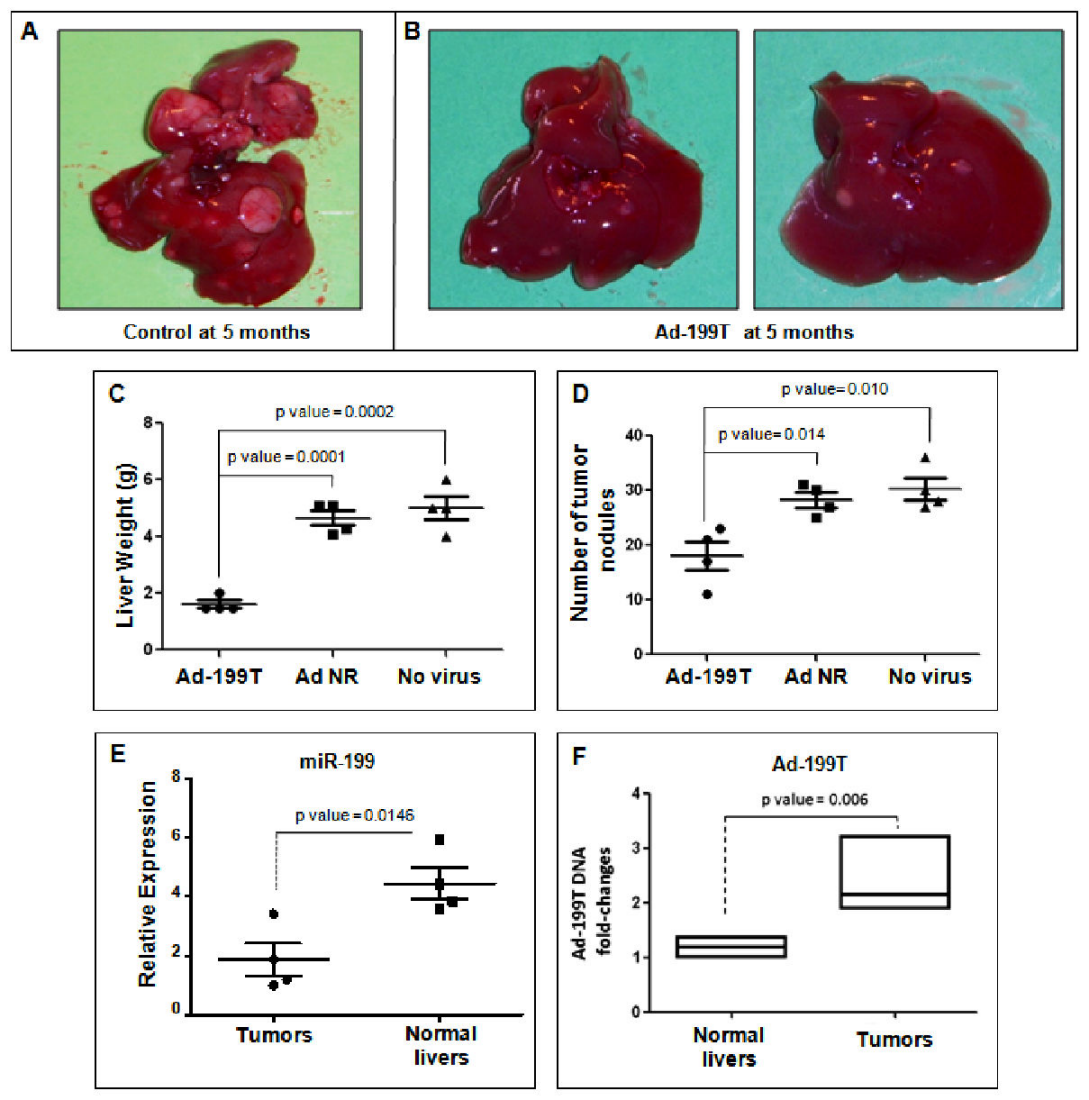
Ad-199T therapeutic activity against DENA-induced tumors in HCC mouse model. A group of TG221 transgenic male mice was treated intra-peritoneum with the carcinogen DEN to boost the development of liver tumors. Three experimental groups, consisting of four mice each, were then defined: the first group was infected two times, at day 60 and 135 after DEN treatment, with 1x10^8^ I.U. of Ad-199T virus, through tail vein injection; the second group was infected with the Ad-Null-Control non-replicative adenovirus (Ad NR), at the same time points with the same I.U; the third group was the not infected control group. All the mice were sacrificed at five months of age and livers collected. (**A**-**B**) Macroscopically, tumors appeared to be less and smaller in mice treated with the Ad-199T virus in comparison with mice either untreated or treated with Ad NR. Quantitative parameters confirmed the qualitative observations. (**C**) Tumor burden was reduced in Ad-199T treated mice as shown by the significant reduction of liver weights. (**D**) The number of tumor nodules was also significantly lower in mice treated with Ad-199T in comparison with the control animals. (**E**) RNAs from normal livers and tumors were analyzed by Real-Time PCR to assess miR-199 levels: as expected tumors displayed a lower expression of miR-199. (**F**) The level of Ad-199T DNA was examined by quantitative Real-Time PCR in normal liver biopsies and tumor nodules of treated mice: a 2-3 fold increase levels in tumor tissues was detected.

After demonstrating the oncolytic activity of Ad-199T in a subcutaneous xenograft tumor model, we aimed at verifying whether Ad-199T could control liver primary tumors. We employed a transgenic mouse model over-expressing microRNA-221 (TG221) [24], which was shown to be highly predisposed to the development of liver tumors and, most importantly, tumors exhibited a miRNA pattern similar to human HCC. Useful for the objective of this study, we previously showed that miR-199 is down-regulated in liver tumors arising in this model. Three experimental groups, consisting of four mice each, were defined: one group was infected with Ad-199T, at day 60 and 135 after DEN injection, each time with 1x10^8^ I.U. of Ad-199T virus, through injection into the tail vein. The other groups were controls: one was infected with a non-replicative adenovirus (Ad NR), to assess the effect of an eventual immune response against liver cells infected by an adenovirus, the other group was a non-infected control, to assess the effect of DEN alone. All mice were sacrificed at 5 months of age. Livers displayed macroscopically evident tumor masses in all animals; however, the Ad-199T infected mice displayed a reduced liver tumor burden, either by visual inspection and by total liver weights in comparison with controls (Figure **8A-C**). Histologic analyses confirmed that the number of tumor nodules was significantly lower in mice treated with Ad-199T (Figure **8D**).

To assess if these findings were related to miR-199 expression, we analyzed the level of the miRNA in normal liver and tumor tissues. Quantitative PCR revealed that miR-199 was down-regulated in tumors in comparison with normal liver (p value = 0.0146) (Figure **8E**). Furthermore, to verify whether Ad-199T could more efficiently replicate in tumor tissues, we injected i.v. Ad-199T (1x10^8^ I.U.) into the tail vein of 5-months old mice-bearing tumors. After 48 h, mice were sacrificed and viral DNA quantified by quantitative PCR in normal (4 biopsies) and tumor tissues (6 different nodules). The level of Ad-199T DNA was 2-3-fold higher in tumor nodules than in normal liver tissues (Figure **8F**). Taken together, these findings can be interpreted as evidence that Ad-199T could replicate more efficiently in tumor tissues, where it can produce cytolysis, and this property was most likely miR-199-dependent.

## Discussion

This work establishes that the presence of miR-199 target sites within the 3’ UTR of E1A gene represents a strategy to generate recombinant adenoviruses with significant oncolytic activity against liver cancer, which exhibits low level of miR-199, coupled with reduced hepatoxicity.

Oncolytic virotherapy emerged as a promising experimental approach to fight cancer. Oncolytic Ad5-based viruses demonstrated efficacy and safety in preclinical [25,26] and clinical trials [27,28,29,30]. The possibility to improve therapeutic efficacy by combining adenoviral virotherapy and chemo or radiation therapy to eradicate malignant glioma produced highly encouraging results with evidence complete tumor eradication in animal models [31,32,33].

For safe and effective gene therapy, target tissue-restricted virus expression is desirable. Conditionally Replicative Adenoviruses (CRAds) specifically aimed at killing tumor cells while sparing normal cells have been developed as new agents for cancer therapy [4,34,35]. Various methods have been used to achieve a selective expression. These approaches included the use of tumor-specific promoters to drive E1A gene expression [7,36,37,38] or the E1B deletion, which restricts the oncolytic activity to p53-defective tumor cells. Among these, ONYX-015 was the first to be tested in clinical trials, revealing itself as a well-tolerated and safe tool and a promising therapeutic agent in cancer [39]. Moreover, its use in combination with standard chemotherapy was demonstrated to increase antitumor activity [40]. By using a very similar virus, H101, a multicenter randomized phase 3 clinical trial showed that the combination therapy yielded a 27% increase in overall response rates compared with fluorouracil plus cisplatin-based chemotherapy alone [41]. This virus was approved by Chinese State Food and Drug Administration for the treatment of late-stage refractory nasopharyngeal cancer.

Here, to improve tumor specificity and reduce toxicity for normal cells, we developed a novel CRAd whose replication was controlled by miR-199. The use of miRNA target (miRT) sequences was first described by Naldini and colleagues to specifically modulate transgene expression in hematopoietic cells [42] or hepatocytes [43]. The present work is the first that make use of miR-199 to target an oncolytic virus to cancer cells *in vivo*. Other works have employed miRNAs to reduce viral pathogenicity in normal tissues [44,45]. Kelly et al. could restrict replication of an oncolytic coxsackievirus (CVA21) to reduce its replication in normal muscle tissue and thus reduce muscle inflammation without compromising tumor cell-killing ability [46]. Fu and colleagues constructed a LCSOV (liver-cancer-specific oncolytic virus) in which the essential viral glycoprotein H gene (*gH gene*) was controlled by both an apoE-AAT liver-specific promoter and the presence of complementary sequences to miR-122, miR-124a and let-7a in its 3’ UTR [47]. Using that strategy they were able to generate an oncolytic virus able to kill HCC cells in a xenograft model.

The Ad-199T virus was designed to replicate in miR-199-negative cells. This objective was achieved by introducing into the viral genome multiple miR-199 target sites able to modulate the expression of the *E1A* gene. Since virtually 100% HCCs exhibit a strong down-regulation of miR-199 which is instead expressed at substantial level in normal hepatocytes, where it represents the third most highly expressed miRNA [48], this virus was designed to prevent cytolytic activity in healthy cells, thereby addressing the important issue of a novel therapeutic approach with improved efficacy and safety in HCC.

By investigating *in vitro* and *in vivo* properties of Ad-199T, we demonstrated that this CRAd could replicate very poorly in cells expressing miR-199, while its replication could proceed regularly in cells lacking the expression of this miRNA. Since miR-199 is highly expressed in normal liver, but not in HCC, this virus seems to be well suited for the treatment of liver cancer. By testing various experimental *in vivo* models, we confirmed this potential opportunity. First, in 3 days old mice, which have a limited or absent immune response [49], Ad-199T was not able to replicate in the liver while an identical control virus, lacking the miR-199 target sites, could efficiently undergo several rounds of replication. In this model, control adenovirus, but not Ad-199T, induced an evident hepatoxicity, as evidenced by histological and immunohistochemical analyses. This foreseeable finding was a consequence of the inhibitory effect imposed by miR-199 on Ad-199T replication, thereby preventing its lytic activity in healthy cells. This property is shared with other recently developed miRNA-dependent oncolytic adenoviruses, like

miR-122-based adenovirus-detargeting vectors, which exhibited a reduced virus-related liver toxicity [9,50,51,52,53]. However, miR-122 is a liver-specific miRNA and is not expressed in any other tissue, leaving open the possibility that toxicities due to viral replication could eventually affect other tissues. Since miR-199 is instead expressed at variable but significant level in any normal tissues, Ad-199T could lack toxicity in tissues other than liver as well.

To support the anti-tumor oncolytic activity of Ad-199T, we proved that the virus could slow-down the growth of xenografts made of liver cancer cells subcutaneously implanted into nude mice. Also in this model, the reduced viral toxicity was supported by the fact that all treated immune deficient mice could survive following 6 consecutive administrations of large amounts of replication-competent viruses. As found with other oncolytic adenoviruses, Ad-199T did not cause tumor regression, but the anti-tumor effect was significant. As previously mentioned, it is possible that this virus, like other CRAds, could find its best use to boost efficacy of chemo or radiotherapy.

We also investigated the anti-tumor effect in an immune-competent host mouse model, highly susceptible to the development of liver primary tumors. Administration of Ad-199T induced a significant reduction of the number and size of tumor nodules, most likely because Ad-199T could replicate more efficiently in neoplastic than in normal liver cells. A conceptually similar but different oncolytic virus was developed by Jin et al. It was engineered as a let-*7* dependent oncolytic adenovirus, able to replicate only in cells lacking the let-*7* miRNA. The authors specify that let-*7* is down-regulated in about 36% of HCC, thereby suggesting that this virus could produce a potential therapeutic effect in this subset of HCCs [54].

Together with the studies on miR-122 and let-*7*, the present study indicates that the knowledge of miRNA expression levels in normal and cancer cells may be applied to the design of oncolytic viruses that combine selective efficacy against cancer cells with minimal adverse toxic effects.

## Supporting Information

Figure S1 miR-199 directly interacts with its target sequence cloned in the pGL3/199T vector, as evaluated by luciferase activity in Hep3B cells. The Firefly Luciferase reporter activity was significantly decreased when pGL3/199T vector was co-transfected with the pre-miR-199a-3p miRNA precursor (p value = 0.007). On the contrary, luciferase activity at the pGL3/199T vector was not significantly affected by a control scramble oligonucleotide. Basal luciferase activity of the pGL3/199T vector is also shown. Untransfected Hep3B cells are indicated as NT. Firefly luciferase activity was normalized on *Renilla* Luciferase activity of the co-transfected pRL-TK vector. Each sample was analyzed in triplicate.
(TIF)

Figure S2 Scheme of Ad-Control and Ad-199T vectors construction. pShuttle/K was the source of E1A/E1B segment, which was joined to the segments IRES (Internal Ribosomal Entry Site) EGFP (Enhanced Green Fluorescent Protein) into the entry vector pENTR11 (Invitrogen) to generate pENTR_E1A/E1B. This latter vector was used as recipient of the miR-199 targeting site (199T) into the MluI restriction site, to generate the pENTR_E1A/199T/E1B vector. Complete adenovirus genomes were produced by site-specific recombination of each entry vector with the destination vector pAd-CMV-V5-Dest (Invitrogen).
(TIF)

Figure S3 HepG2/199 cell line stably express miR-199. The pIRES-miR199 vector, expressing miR-199, was stably transfected in the hepatocellular carcinoma derived cell line HepG2, generating the HepG2/199 cell line. TaqMan, Real Time PCR analysis showed that miR-199 expression was significantly increased in the HepG2/199 cell line in comparison with the basal expression level in the HepG2 cells (p-value = 0.0005) and not significantly different from human normal liver (NL) expression levels (p-value = 0.06). Each sample was analyzed in triplicate.
(TIF)

Figure S4 Histopathology and phospho-H2AX staining in livers infected with Ad-Control or Ad-199T. (**A**) In Ad-Control infected livers, macro-vesicular steatosis associated with disruption of the normal liver architecture can be seen; nuclei are displaced at the edge of the cells by the large fat vacuoles. (**B**) Another feature seen in Ad-Control infected livers was the accumulation of micro-vesicles in the cytoplasm of hepatocytes, which were variable in size with heterogeneous nuclei. (**C**) These histopathology changes were nearly absent in the livers of Ad-199T treated mice. Cell plate structure was conserved, hepatocyte cytoplasm was not generally vacuolated and nuclei showed a very little polymorphism. (**D**) The livers from control mice exhibited very few hepatocytes that stained positive for phospho-H2AX (red arrows). A very faint staining was observed in the nuclei of endothelial cells surrounding hepatic veins (orange arrows). (**E**) Few hepatocytes with apoptotic appearance stained positive for phospho-H2AX (red arrows). In spite of the absence of histopathological changes, some hepatocytes exhibited a faint nuclear staining for phospho-H2AX (blue arrows). (**F**) Livers infected with Ad-Control displayed a nearly ubiquitous IHC staining for phospho-H2AX, detectable in the nuclei of hepatocytes, of endothelial cells and of bile ducts. Apoptotic hepatocytes in the context of necrotic areas show an intense staining for phospho-H2AX (red arrows).
(TIF)

Figure S5 HepLuc cell line stably express Luciferase gene. HepG2 cell line was stably transfected with pIRES-Luc, a vector expressing the Luciferase reporter gene under the control of a CMV promoter. Several HepLuc stable clones were obtained and the reporter gene expression was tested by a Luciferase assay. Each sample was analyzed in triplicate.
(TIF)

Figure S6 Ad-199T and Ad-Control can eliminate implanted tumor cells *in vivo*. Treated animals described in Figure 5 were sacrificed 72 hours after virus injection and the livers were collected (**A**-**C**). Images of the livers showed the presence of tumor masses corresponding to luminescent signal detected at the IVIS luminometer. Tumor masses were larger in uninfected controls and significantly reduced in mice treated with both Ad-199T and Ad-Control viruses.
(TIF)

Figure S7 Evidence of human genomic DNA in mice tumor masses. Genomic DNA was extracted both from normal livers (NL) and tumor masses (Tumor) of mice injected intra-hepatically with HepLuc cells and treated with Ad-199T virus. The mice were sacrificed after 24 and 48 hours. All the samples were analyzed by analytical PCR using primers for the human TPEF (*transmembrane protein containing epidermal growth factor and follistatin domain*) gene. As housekeeping gene, specific primers for mouse β-actin were used. As a negative control (-), mouse tail genomic DNA was used. As a positive control (+), HepG2 genomic DNA was used.
(TIF)

Figure S8 Tumor cell removal is triggered by viral lytic cycle as well as immune response to viral antigens. (**A**) 1x10^6^ HepLuc cells were intra-hepatically implanted into B6D2 wild type mice at 3 days of age. Two hours after cells implantation, mice were examined at the In Vivo Imaging System (IVIS) to verify homogeneity among the various implants. Bioluminescence intensity, measured as luciferase activity is shown as pseudo-color images and is proportional to the amount of tumor cells. The day after, three experimental groups, consisting of six mice each, were defined: one was intra-hepatically injected with 1x10^8^ I.U. of the Ad-199T virus; the second with 1x10^8^ I.U. of a not replicative adenovirus (Ad-NR); the third received no virus. Mice were then monitored at 24h (**B**), 48h (**C**) and 72h (**D**) after virus injection. (**E**) Faster reduction of implanted tumor cells was detected in the Ad-199T virus group than in the not replicative adenovirus or the no virus group. Quantitative photon analysis showed a significant difference (24h, p value = 0.0008; 48h, p value = 0.025; 72h, p value = 0.022) of luminescence in mice treated with Ad-199T versus mice treated with a replicative-defective adenovirus. The difference between the no virus and the not replicative adenovirus groups was significant at 48h and 72h.
(TIF)

Figure S9 Ad-199T replicates in Hep3B cell line. (**A**) To asses miR-199 expression levels in Hep3B cell line, a TaqMan, Real Time PCR was performed. The results showed that Hep3B displayed a very low basal miR-199 expression level, even lower than HepG2 cells. Each sample was analyzed in triplicate. (**B**-**C**) To verify miR-199-depentent replication capability of Ad-199T virus in Hep3B cells, cells were seeded and infected with 1x10^6^ I.U of Ad-199T and harvested after 24, 48, 72, 96 and 120hrs. Genomic DNAs extracted were analyzed by analytical and quantitative PCR as a fold change copies of Adeno DNA referred to the lower level of Adenovirus copies.
(TIF)
