# Supplementary figures and images for: Anti-Tumor Activity of a miR-199-dependent Oncolytic Adenovirus
Source: PLoS One. 2013 Sep 12;8(9):e73964. doi: 10.1371/journal.pone.0073964 (PMC3771938; doi:10.1371/journal.pone.0073964)

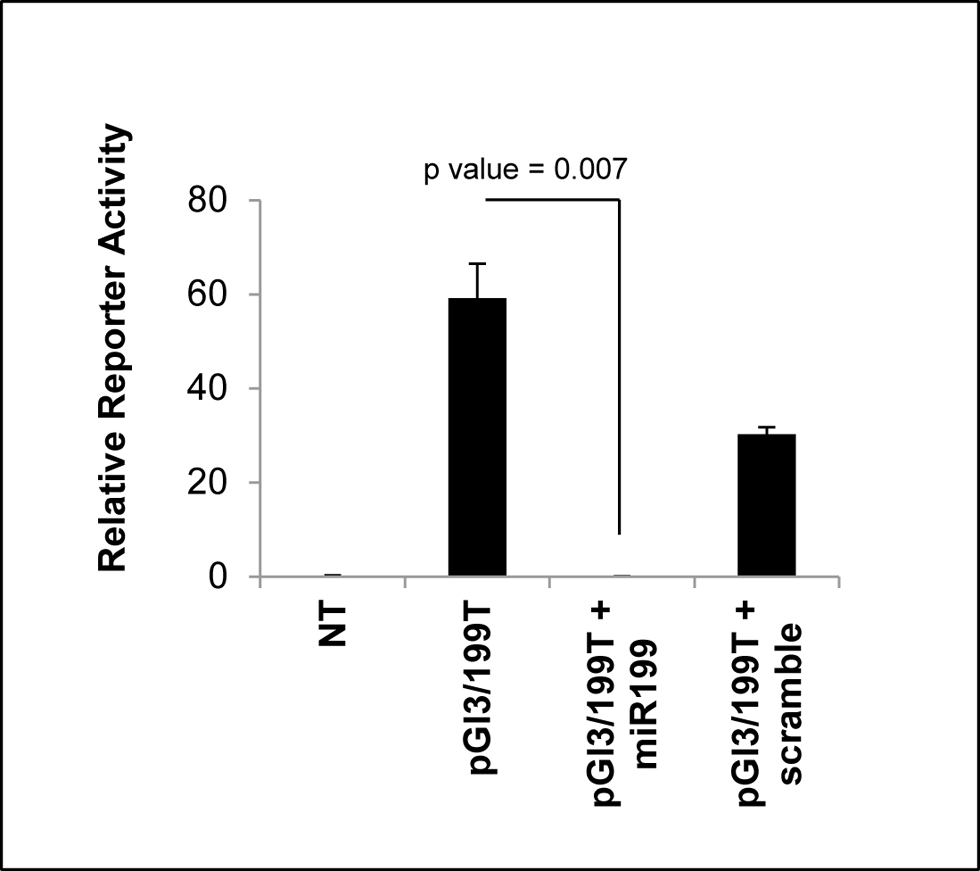

Supplement: Figure S1 — The Firefly Luciferase reporter activity was significantly decreased when pGL3/199T vector was co-transfected with the pre-miR-199a-3p miRNA precursor (p value = 0.007). On the contrary, luciferase activity at the pGL3/199T vector was not significantly affected by a control scramble oligonucleotide. Basal luciferase activity of the pGL3/199T vector is also shown. Untransfected Hep3B cells are indicated as NT. Firefly luciferase activity was normalized on Renilla Luciferase activity of the co-transfected pRL-TK vector. Each sample was analyzed in triplicate. (TIF) [file pone.0073964.s001.tif]

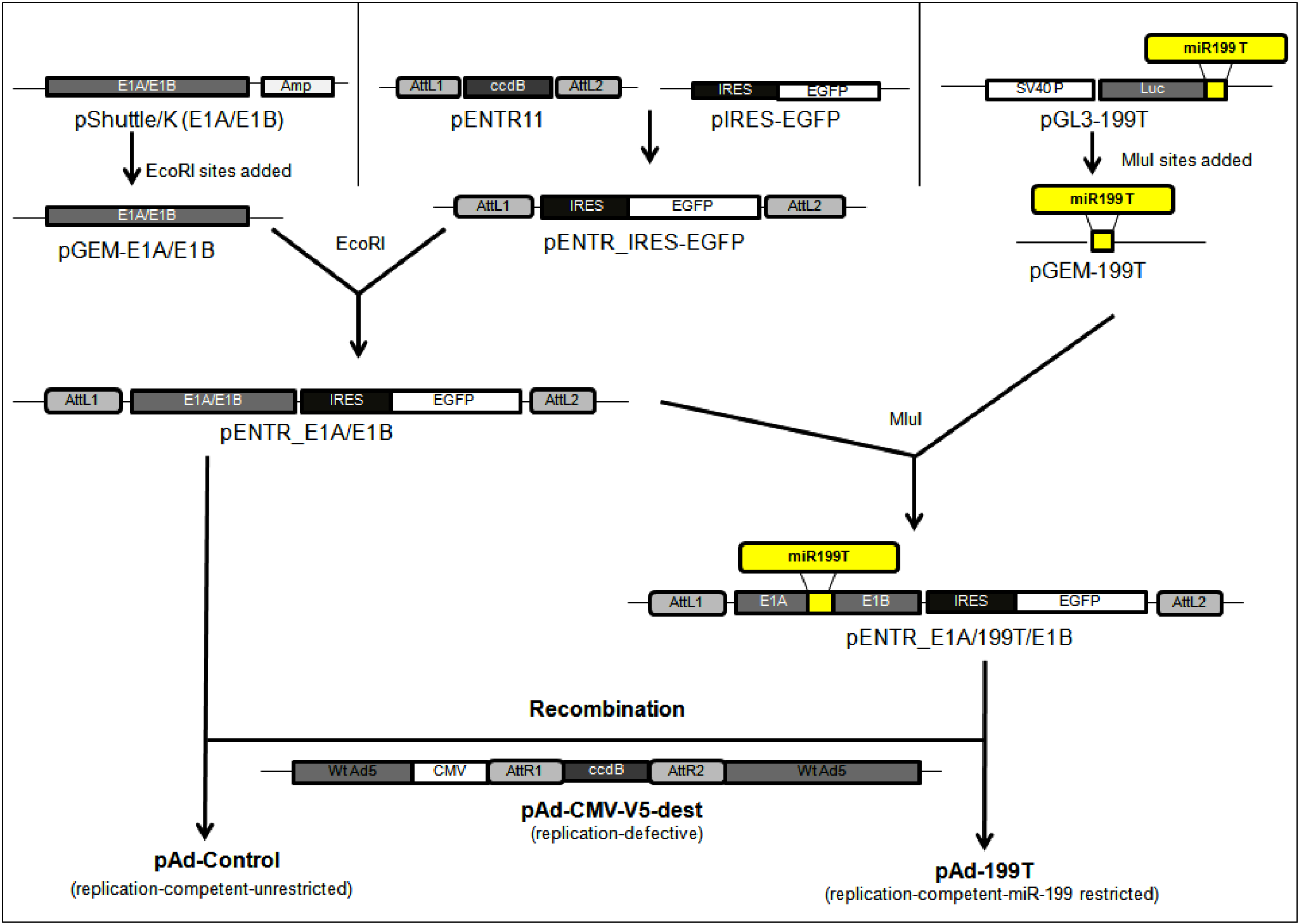

Supplement: Figure S2 — pShuttle/K was the source of E1A/E1B segment, which was joined to the segments IRES (Internal Ribosomal Entry Site) EGFP (Enhanced Green Fluorescent Protein) into the entry vector pENTR11 (Invitrogen) to generate pENTR_E1A/E1B. This latter vector was used as recipient of the miR-199 targeting site (199T) into the MluI restriction site, to generate the pENTR_E1A/199T/E1B vector. Complete adenovirus genomes were produced by site-specific recombination of each entry vector with the destination vector pAd-CMV-V5-Dest (Invitrogen). (TIF) [file pone.0073964.s002.tif]

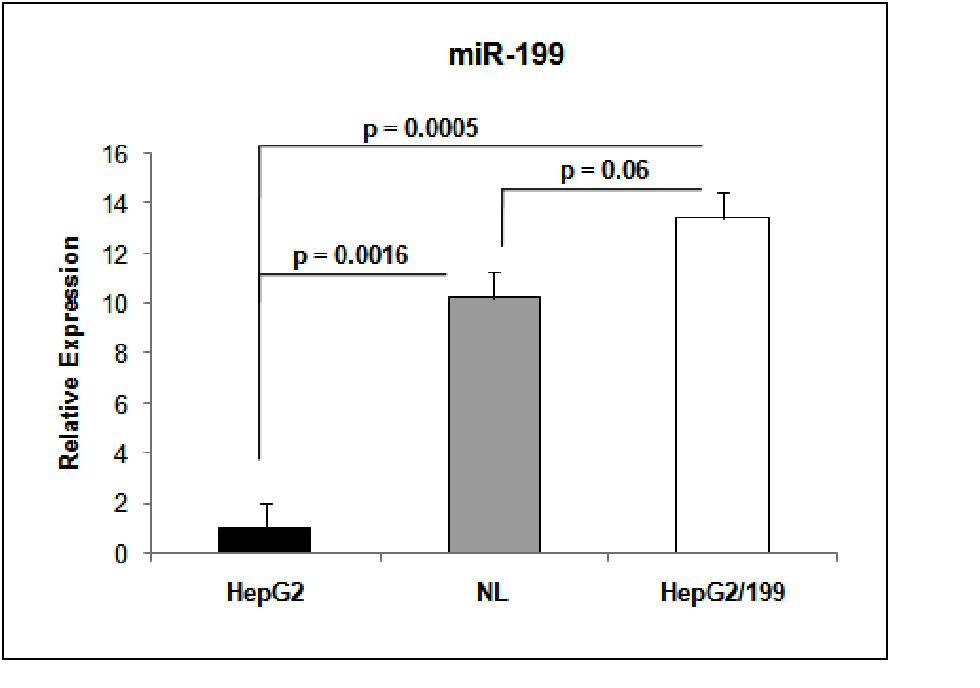

Supplement: Figure S3 — The pIRES-miR199 vector, expressing miR-199, was stably transfected in the hepatocellular carcinoma derived cell line HepG2, generating the HepG2/199 cell line. TaqMan, Real Time PCR analysis showed that miR-199 expression was significantly increased in the HepG2/199 cell line in comparison with the basal expression level in the HepG2 cells (p-value = 0.0005) and not significantly different from human normal liver (NL) expression levels (p-value = 0.06). Each sample was analyzed in triplicate. (TIF) [file pone.0073964.s003.tif]

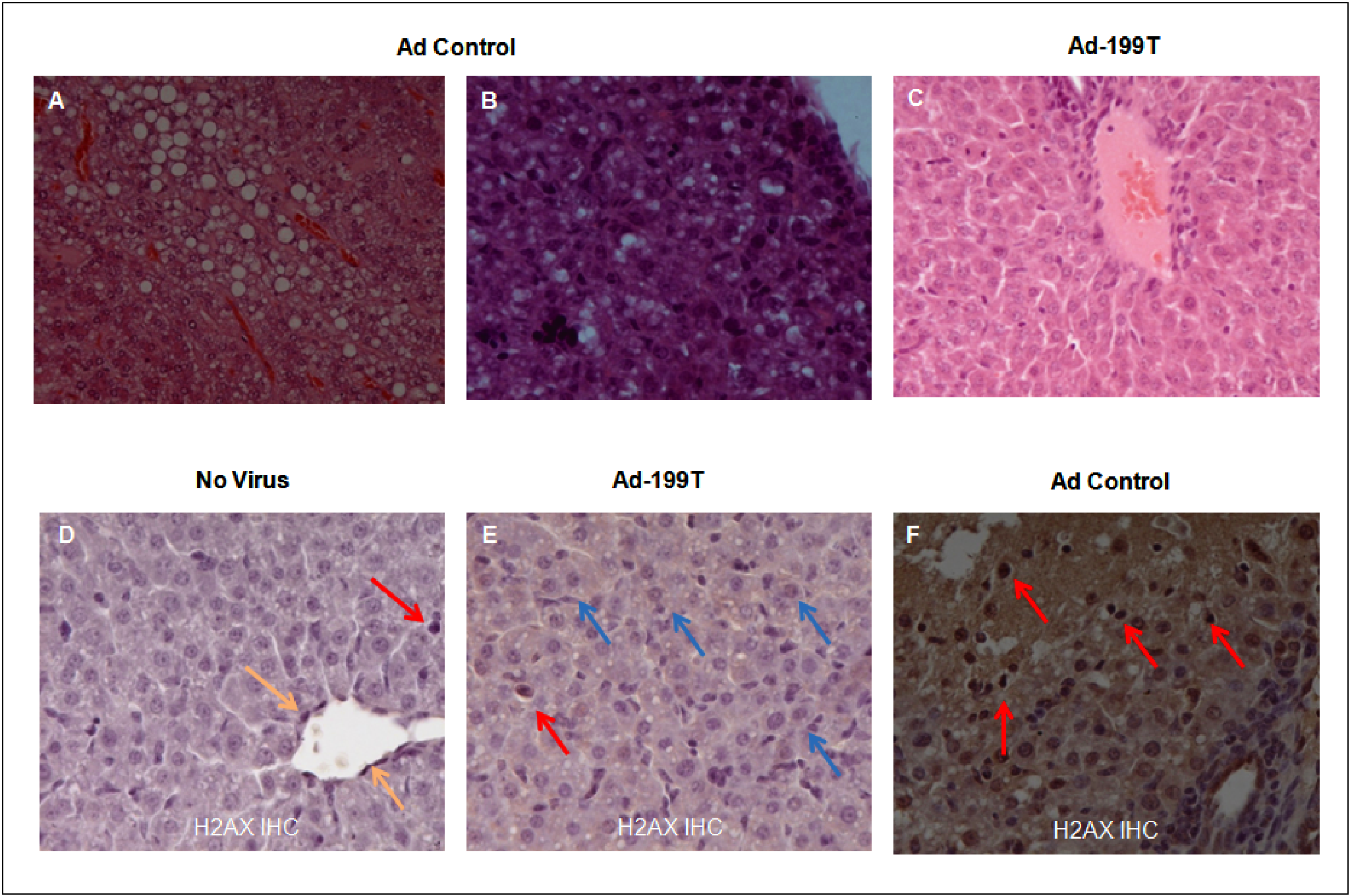

Supplement: Figure S4 — (A) In Ad-Control infected livers, macro-vesicular steatosis associated with disruption of the normal liver architecture can be seen; nuclei are displaced at the edge of the cells by the large fat vacuoles. (B) Another feature seen in Ad-Control infected livers was the accumulation of micro-vesicles in the cytoplasm of hepatocytes, which were variable in size with heterogeneous nuclei. (C) These histopathology changes were nearly absent in the livers of Ad-199T treated mice. Cell plate structure was conserved, hepatocyte cytoplasm was not generally vacuolated and nuclei showed a very little polymorphism. (D) The livers from control mice exhibited very few hepatocytes that stained positive for phospho-H2AX (red arrows). A very faint staining was observed in the nuclei of endothelial cells surrounding hepatic veins (orange arrows). (E) Few hepatocytes with apoptotic appearance stained positive for phospho-H2AX (red arrows). In spite of the absence of histopathological changes, some hepatocytes exhibited a faint nuclear staining for phospho-H2AX (blue arrows). (F) Livers infected with Ad-Control displayed a nearly ubiquitous IHC staining for phospho-H2AX, detectable in the nuclei of hepatocytes, of endothelial cells and of bile ducts. Apoptotic hepatocytes in the context of necrotic areas show an intense staining for phospho-H2AX (red arrows). (TIF) [file pone.0073964.s004.tif]

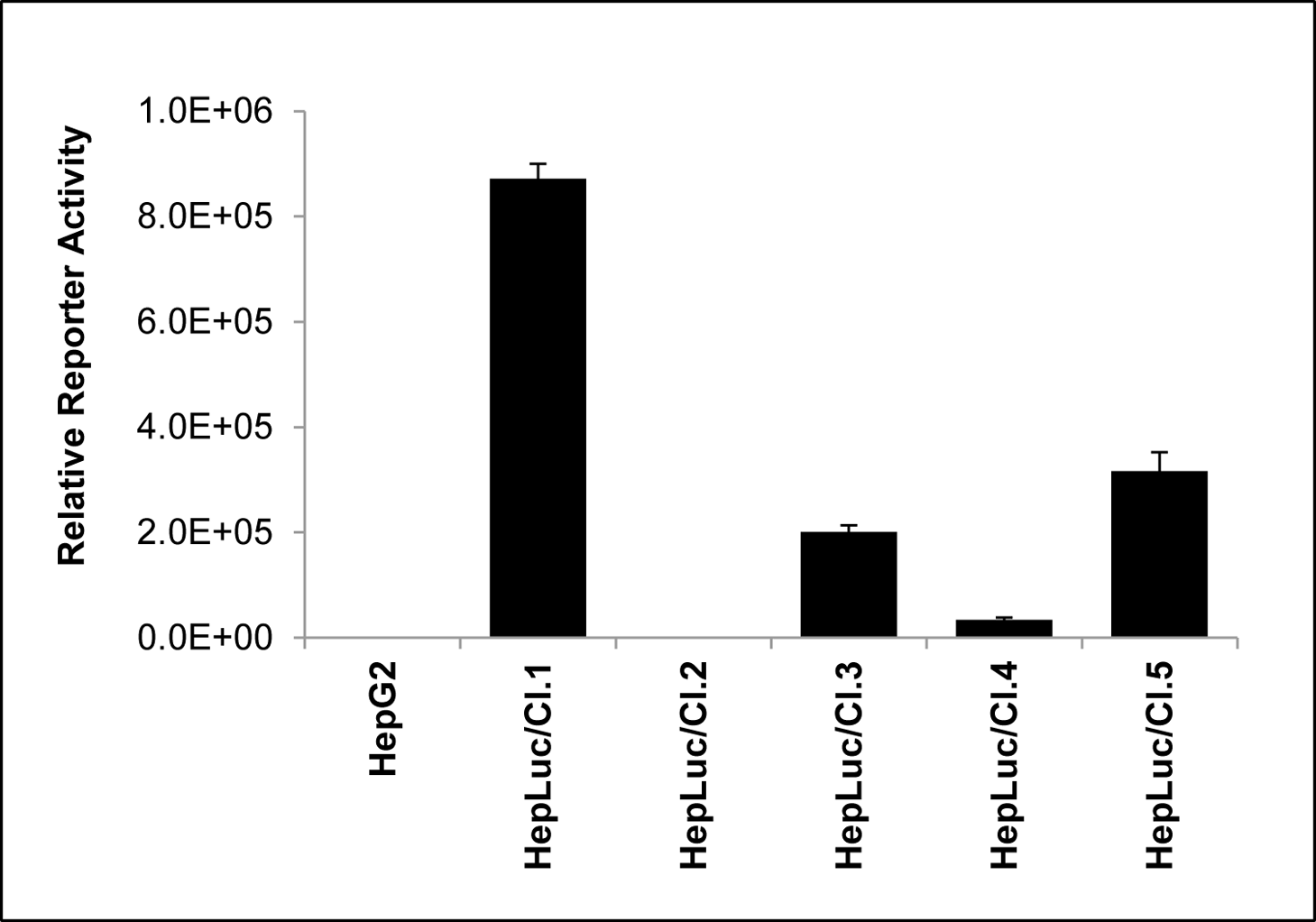

Supplement: Figure S5 — HepG2 cell line was stably transfected with pIRES-Luc, a vector expressing the Luciferase reporter gene under the control of a CMV promoter. Several HepLuc stable clones were obtained and the reporter gene expression was tested by a Luciferase assay. Each sample was analyzed in triplicate. (TIF) [file pone.0073964.s005.tif]

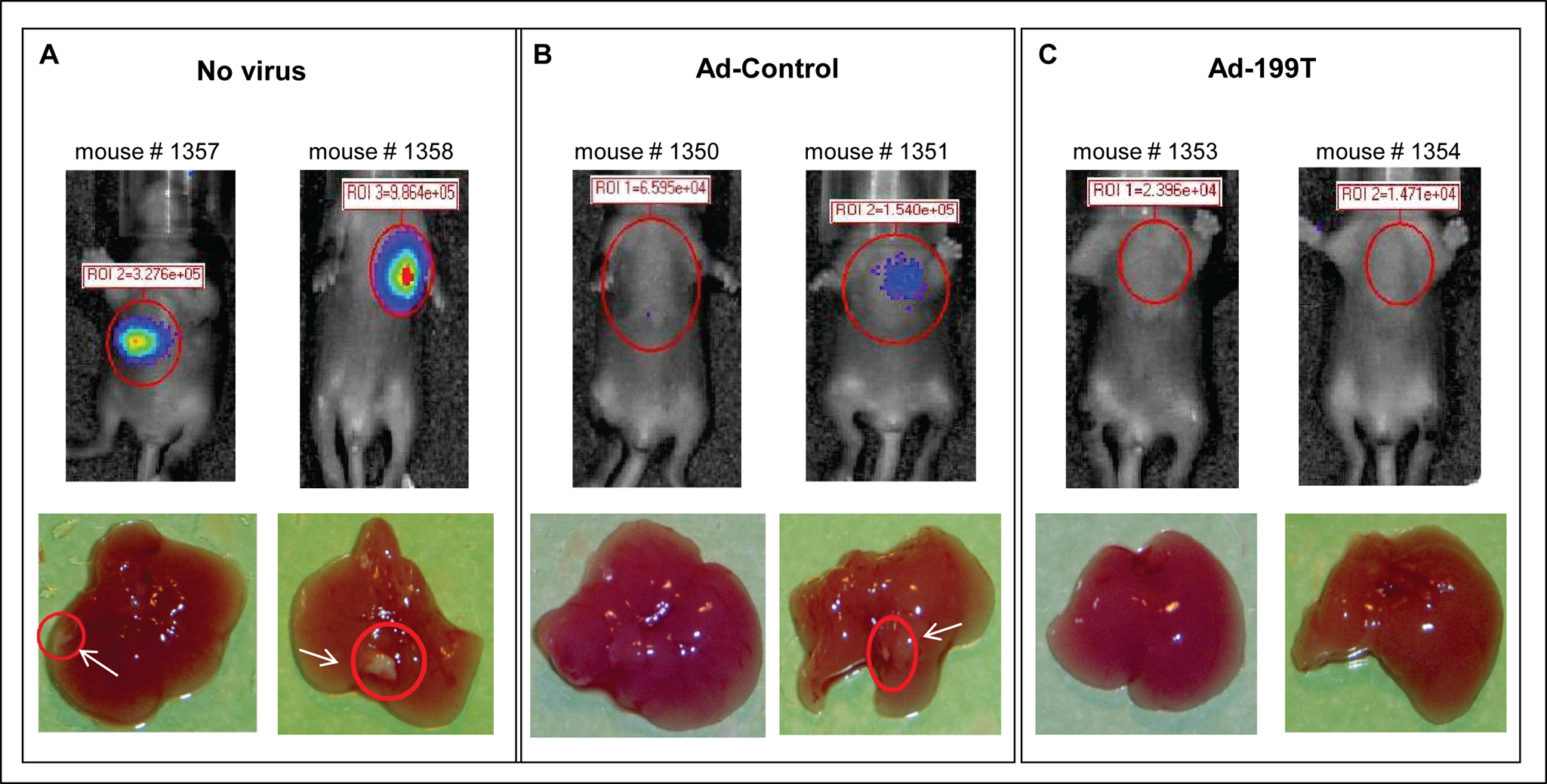

Supplement: Figure S6 — Treated animals described in Figure 5 were sacrificed 72 hours after virus injection and the livers were collected (A-C). Images of the livers showed the presence of tumor masses corresponding to luminescent signal detected at the IVIS luminometer. Tumor masses were larger in uninfected controls and significantly reduced in mice treated with both Ad-199T and Ad-Control viruses. (TIF) [file pone.0073964.s006.tif]

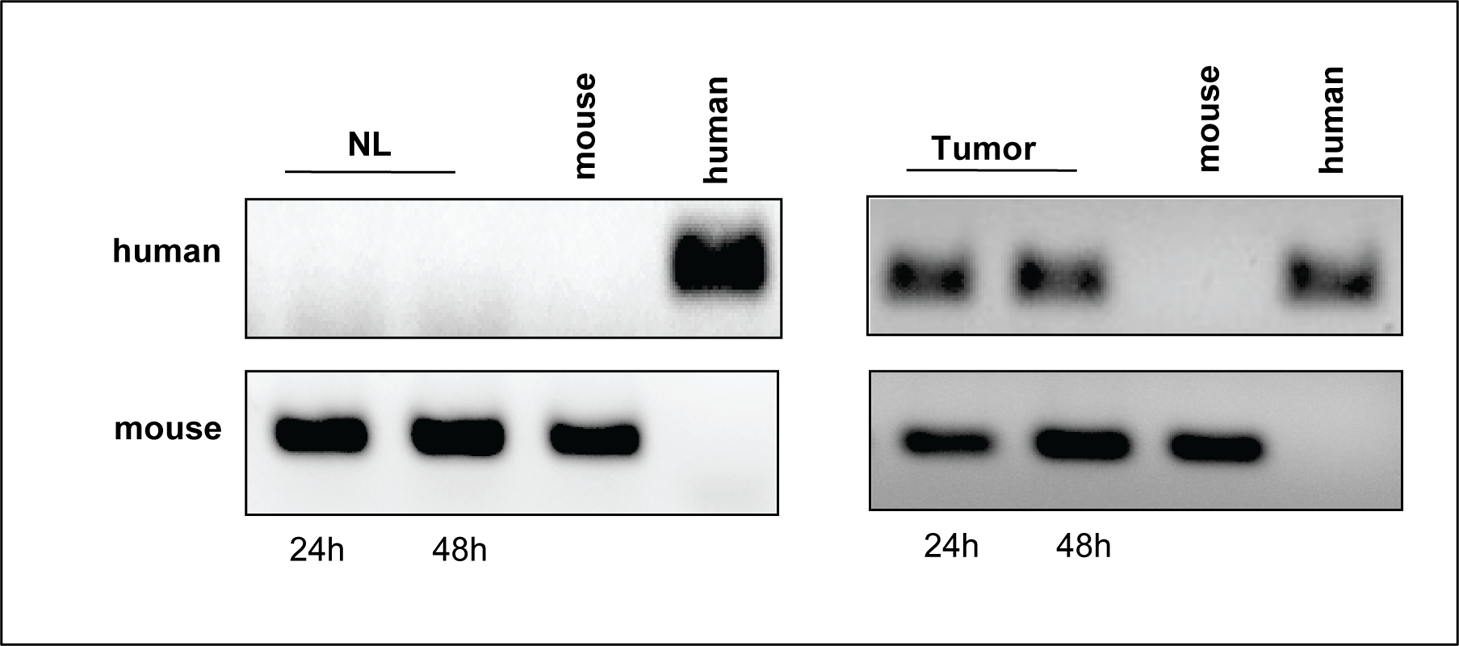

Supplement: Figure S7 — Genomic DNA was extracted both from normal livers (NL) and tumor masses (Tumor) of mice injected intra-hepatically with HepLuc cells and treated with Ad-199T virus. The mice were sacrificed after 24 and 48 hours. All the samples were analyzed by analytical PCR using primers for the human TPEF (transmembrane protein containing epidermal growth factor and follistatin domain) gene. As housekeeping gene, specific primers for mouse β-actin were used. As a negative control (-), mouse tail genomic DNA was used. As a positive control (+), HepG2 genomic DNA was used. (TIF) [file pone.0073964.s007.tif]

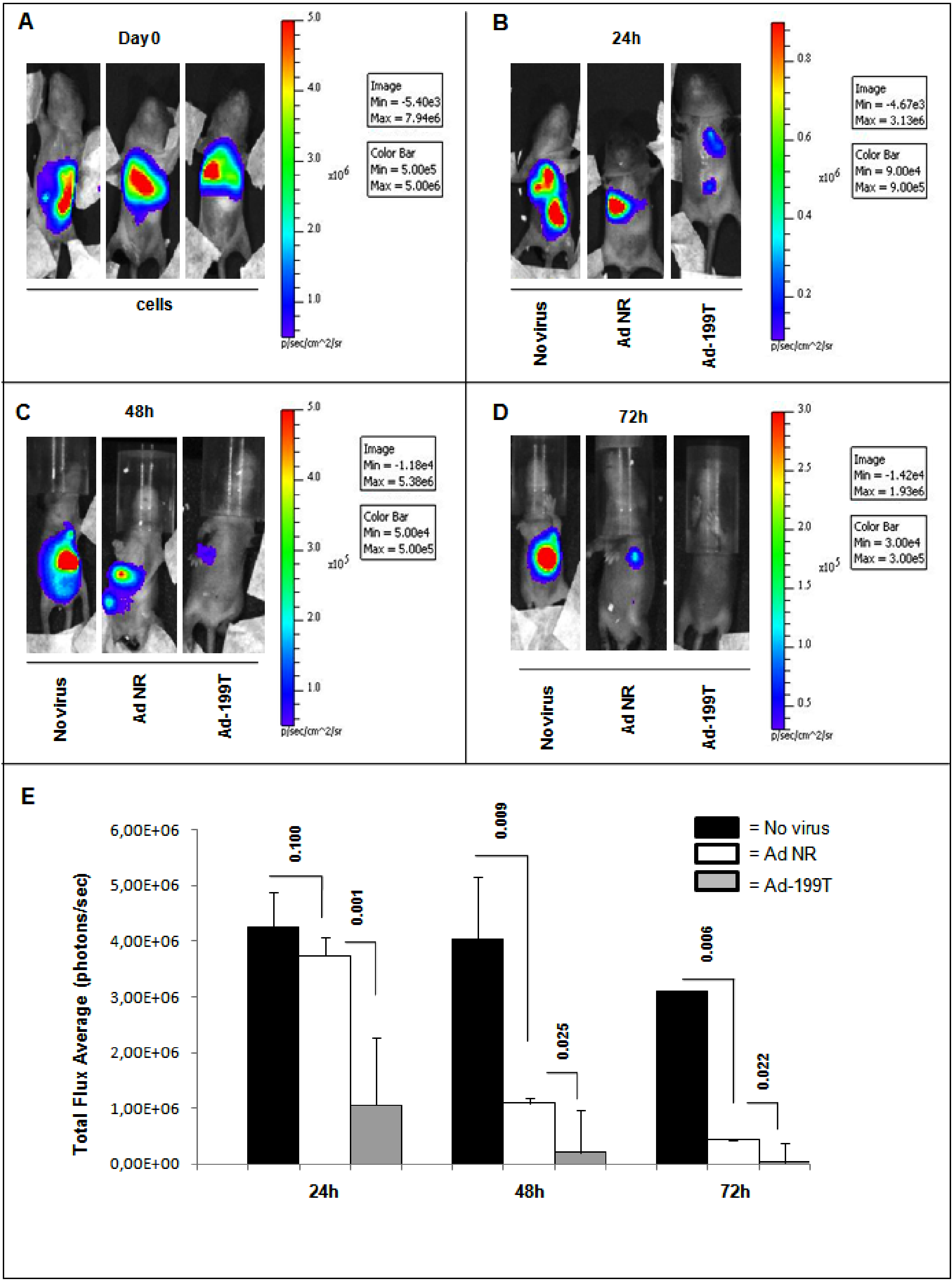

Supplement: Figure S8 — (A) 1x10^6^ HepLuc cells were intra-hepatically implanted into B6D2 wild type mice at 3 days of age. Two hours after cells implantation, mice were examined at the In Vivo Imaging System (IVIS) to verify homogeneity among the various implants. Bioluminescence intensity, measured as luciferase activity is shown as pseudo-color images and is proportional to the amount of tumor cells. The day after, three experimental groups, consisting of six mice each, were defined: one was intra-hepatically injected with 1x10^8^ I.U. of the Ad-199T virus; the second with 1x10^8^ I.U. of a not replicative adenovirus (Ad-NR); the third received no virus. Mice were then monitored at 24h (B), 48h (C) and 72h (D) after virus injection. (E) Faster reduction of implanted tumor cells was detected in the Ad-199T virus group than in the not replicative adenovirus or the no virus group. Quantitative photon analysis showed a significant difference (24h, p value = 0.0008; 48h, p value = 0.025; 72h, p value = 0.022) of luminescence in mice treated with Ad-199T versus mice treated with a replicative-defective adenovirus. The difference between the no virus and the not replicative adenovirus groups was significant at 48h and 72h. (TIF) [file pone.0073964.s008.tif]

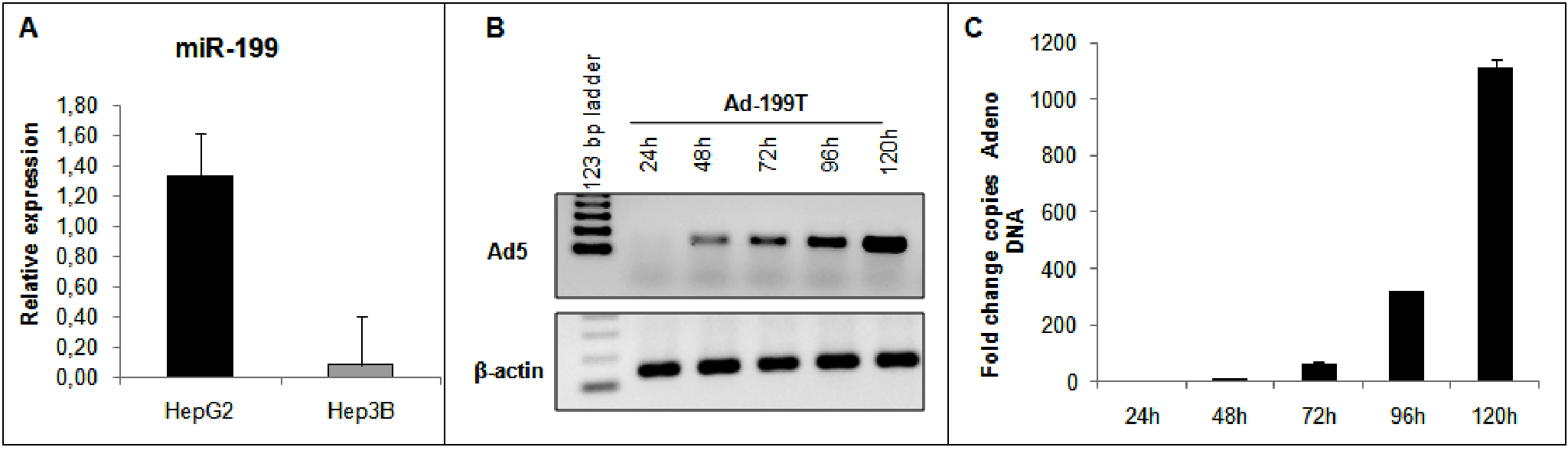

Supplement: Figure S9 — (A) To asses miR-199 expression levels in Hep3B cell line, a TaqMan, Real Time PCR was performed. The results showed that Hep3B displayed a very low basal miR-199 expression level, even lower than HepG2 cells. Each sample was analyzed in triplicate. (B-C) To verify miR-199-depentent replication capability of Ad-199T virus in Hep3B cells, cells were seeded and infected with 1x10^6^ I.U of Ad-199T and harvested after 24, 48, 72, 96 and 120hrs. Genomic DNAs extracted were analyzed by analytical and quantitative PCR as a fold change copies of Adeno DNA referred to the lower level of Adenovirus copies. (TIF) [file pone.0073964.s009.tif]
